# Hepatitis B Virus Induces IL-23 Production in Antigen Presenting Cells and Causes Liver Damage via the IL-23/IL-17 Axis

**DOI:** 10.1371/journal.ppat.1003410

**Published:** 2013-06-27

**Authors:** Qinghong Wang, Jijun Zhou, Bei Zhang, Zhiqiang Tian, Jun Tang, Yanhua Zheng, Zemin Huang, Yi Tian, Zhengcai Jia, Yan Tang, Jennifer C. van Velkinburgh, Qing Mao, Xiuwu Bian, Yifang Ping, Bing Ni, Yuzhang Wu

**Affiliations:** 1 Institute of Immunology, Third Military Medical University, Chongqing, People′s Republic of China; 2 Ministry of Education Key Laboratory of Child Development and Disorders, Pediatric Research Institute, Children's Hospital of Chongqing Medical University, Chongqing, People′s Republic of China; 3 Department of Infectious Diseases, Southwestern Hospital, Third Military Medical University, Chongqing, People′s Republic of China; 4 Department of Immunology, Medical College of Qingdao University, Qingdao, People′s Republic of China; 5 van Velkinburgh Initiative for Collaboratory Biomedical Research, Santa Fe, New Mexico, United States of America; 6 Department of Pathology, Southwestern Hospital, Third Military Medical University, Chongqing, People′s Republic of China; University of California, San Diego, United States of America

## Abstract

IL-23 regulates myriad processes in the innate and adaptive immune systems, and is a critical mediator of the proinflammatory effects exerted by Th17 cells in many diseases. In this study, we investigated whether and how hepatitis B virus (HBV) causes liver damage directly through the IL-23 signaling pathway. In biopsied liver tissues from HBV-infected patients, expression of both IL-23 and IL-23R was remarkably elevated. *In vivo* observations also indicated that the main sources of IL-23 were myeloid dendritic cells (mDCs) and macrophages. Analysis of *in vitro* differentiated immature DCs and macrophages isolated from healthy donors revealed that the HBV surface antigen (HBsAg) efficiently induces IL-23 secretion in a mannose receptor (MR)-dependent manner. Culture with an endosomal acidification inhibitor and the dynamin inhibitor showed that, upon binding to the MR, the HBsAg is taken up by mDCs and macrophages through an endocytosis mechanism. In contrast, although the HBV core antigen (HBcAg) can also stimulate IL-23 secretion from mDCs, the process was MR- and endocytosis-independent. In addition, IL-23 was shown to be indispensible for HBsAg-stimulated differentiation of naïve CD4^+^ T cells into Th17 cells, which were determined to be the primary source of IL-17 in HBV-infected livers. The cognate receptor, IL-17R, was found to exist on the hepatic stellate cells and mDCs, both of which might represent the potential target cells of IL-17 in hepatitis B disease. These data provide novel insights into a yet unrecognized mechanism of HBV-induced hepatitis, by which increases in IL-23 expression, through an MR/endocytosis-dependent or -independent manner, produce liver damage through the IL-23/IL-17 axis.

## Author Summary

While it is known that IL-23 plays a pivotal role in maintenance of the Th17 phenotype and their production of the IL-17 cytokine, the mechanisms by which HBV induces particular immune cell types to produce IL-23 remain unknown. In the study of human hepatitis B described herein, we demonstrated that IL-23 is principally derived from the liver myeloid dendritic cells (mDCs) and macrophages. *In vitro* assay showed that mDCs produce large amounts of IL-23 upon stimulation with HBV surface antigen (HBsAg) through the mannose receptor (MR) and an endocytosis mechanism. In contrast, although the HBV core antigen (HBcAg) was also capable of stimulating IL-23 secretion from mDCs, the process occurs in an MR- and endocytosis-independent manner. IL-23 was also shown to efficiently stimulate the differentiation of naïve CD4^+^ T cells into Th17 cells in the presence of HBsAg or HBcAg; furthermore, the Th17 cells were shown to be the primary source of IL-17. The results also indicated that both hepatic satellite cells and mDCs might be the potential target cells of IL-17 in hepatitis B disease. Therefore, our study not only provides further insights into the mechanisms underlying HBV pathogenesis, but also suggests the potential intervening targets for patient treatment.

## Introduction

Hepatitis B virus (HBV) is a noncytopathic, hepatotropic and stealth DNA virus that has been implicated in the etiology of chronic hepatitis B (CHB) and HBV-associated acute-on-chronic liver failure (ACLF). HBV-induced hepatic injury is known to be mediated by a variety of immunocytes that play important roles in the development and progression of hepatitis B. Among these host immune cells, the T cells are considered the main effector cells contributing to the pathogenesis of hepatitis B disease. Furthermore, the CD4^+^ and CD8^+^ T cell subpopulations have both been shown to play key roles in antiviral defenses, as well as in the hepatocellular damage that accompanies hepatitis B viral infection [1].

CD4^+^CD25^+^Foxp3^+^ regulatory T (Treg) cells are a subset of the CD4^+^ T cells that function as inhibitors of T cell-mediated responses. While this action ameliorates T cell-mediated hepatocellular damage, it also creates an environment favorable to persistent hepatitis viral infection and tumor formation [2], [3]. More recently, however, it has been reported that HBV-infected patients have a remarkably high amount of another subset of T helper (Th) cells, the interleukin (IL)-17-secreting Th17 cells, which have been verified as closely associated with the development and severity of liver damage in patients with hepatitis B [4]–[6] and HBV-related liver fibrosis [7], [8]. However, the detailed mechanisms for the roles of Th17 cells in hepatitis B disease remain to be fully elucidated.

It has been demonstrated that, in hepatitis B patients, the Th17 cell-associated liver inflammation is positively associated with IL-23 cytokine level, as described in our previous review [6]. IL-23, a member of the IL-12 family, has been shown to regulate a myriad of processes in the innate and adaptive immune systems [9], [10], including promoting the antigen presentation abilities of antigen presenting cells (APCs) and enhancing the effector function of T cells [11]. In our previous experiment, we found that the mRNA expressions of IL-23 were enhanced in the liver tissue of hepatitis B patients [12]; recent study further indicated that IL-23 plays a pathological role through IL-17 production in Concanavalin A (Con A)-induced hepatitis [13]. All these data suggest that IL-23 might play an important role in the pathogenesis of hepatitis B. However, the precise source cells of IL-23, the mechanism by which HBV induces their production of IL-23, and the concrete functions of IL-23 in hepatitis B patients have to be clarified in order to gain an accurate and useful understanding of the immunological mechanisms underlying HBV infection.

IL-23R has been demonstrated to be mainly expressed on Th17 cells (interleukin-17-producing CD4^+^ T cells) and IL-23 is thought to play a pivotal role in the differentiation and maintenance of function of Th17 cells [14], [15]. Recent research indicated that a remarkable amount of Th17 cells infiltrate the liver tissue in hepatitis B patients and the extent of Th17 infiltration correlated with severity of liver damage [4], [16], [17]. IL-17 must bind to its cognate receptor (IL-17R) to fulfill its functions, such as inducing the release of other cytokines that lead to proinflammatory processes and neutrophil-mobilization [14], [18]. In Con A-induced hepatitis [19], IL-17R was found to be highly expressed on Kupffer cells and the IL-17/IL-17R signaling pathway was characterized as critically involved in the pathogenesis. Zhang *et al.* reported that IL-17R was uniquely expressed on monocytes and myeloid dendritic cells (mDCs) from the peripheral blood mononuclear cell (PBMC) population of CHB patients [4]. However, the cell type source of IL-17R in HBV-infected liver tissue remains unknown, and may involve any one or multiple types of the immune cells known to reside in the diseased organ, including lymphocytes, neutrophils, mDCs, or/and plasmacytoid dendritic cells (pDCs) [20], [21].

Herein, we investigated the role of the IL-23/IL-17 axis in HBV-infected humans by using human liver biopsy samples. We found that, upon binding to the mannose receptor (MR) on mDCs and macrophages, the hepatitis B surface antigen (HBsAg) could be taken up through an endocytosis mechanism, followed by efficient IL-23 secretion from these cells. In contrast to HBsAg, HBV core antigen (HBcAg) could also stimulate mDCs and macrophages to produce IL-23, but in an MR- and endocytosis-independent manner. Clinical observation further demonstrated that IL-23 expressions were significantly up-regulated in hepatitis B patients, and identified mDCs and macrophages as the main sources of IL-23. In response to the elevated IL-23 level, Th17 cells were found to be stimulated to produce large amounts of IL-17, which would subsequently contribute to the pathogenesis of hepatitis B disease. We also found that the key Treg cell functional cytokine, IL-10, and not TGF-β, could markedly inhibit the HBsAg-stimulated IL-23 production from mDCs *in vitro*. Thus, the findings from this study not only provide novel insights into the mechanisms of the IL-23/IL-17 axis, supporting the pathogenesis of HBV infection, but also identify potential targets of interventional strategies for treating hepatitis B patients through manipulation of the IL-23/IL-17 axis.

## Results

### Hepatic IL-23 expression is significantly elevated in HBV infected patients and mainly derived from APCs

**Figure 1:**
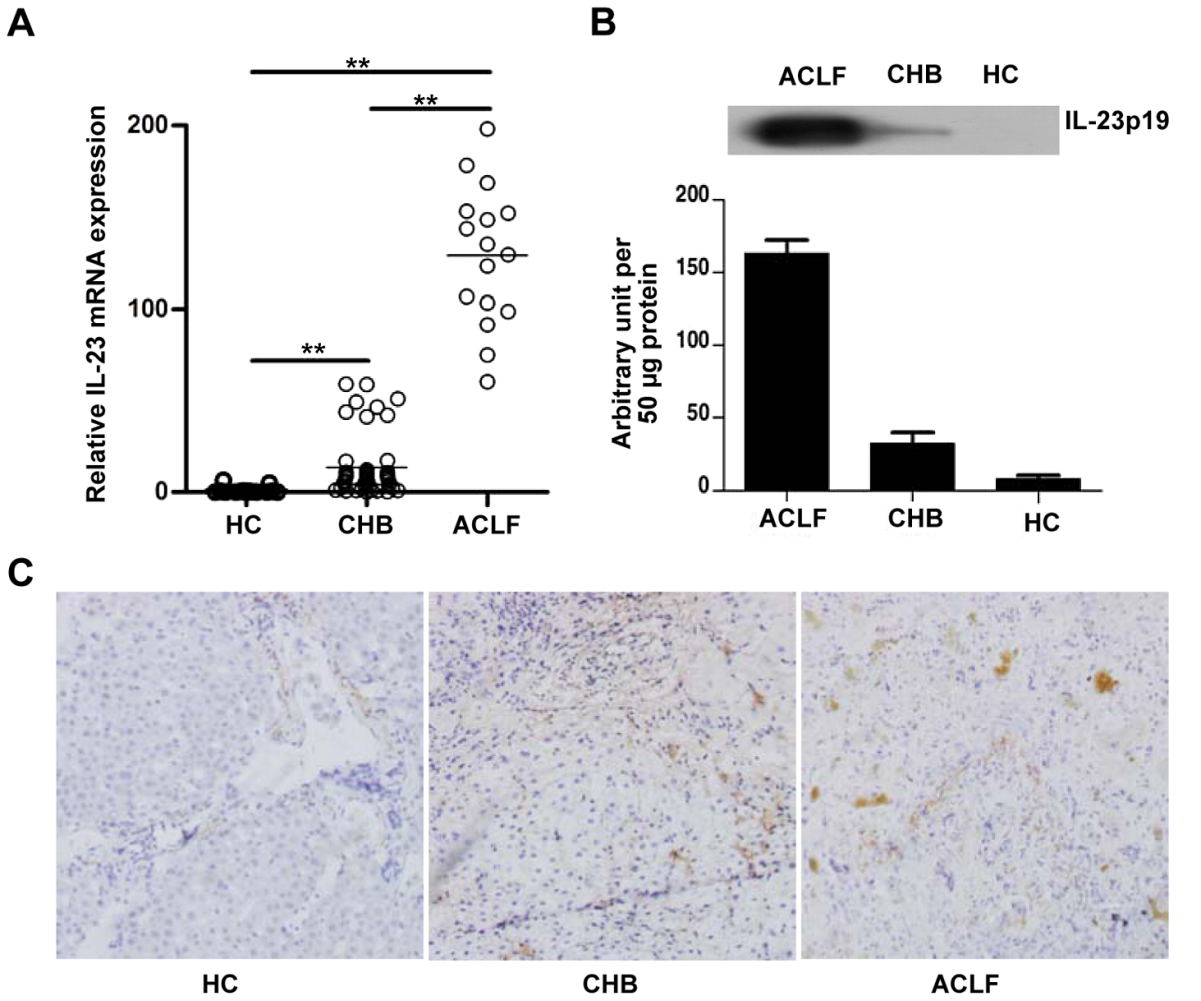
IL-23 expression is increased in hepatitis B liver tissue. (**A**) Relative mRNA expression of IL-23 in liver tissue of various groups was analyzed by qPCR. (**B**) Protein expression of IL-23 was analyzed by Western blot assay. Error bars indicate SD. *P<0.05; **P<0.01. (**C**) *In situ* expression of IL-23 in liver tissues was detected by immunohistochemical staining (magnification 100×).

IL-23 has been demonstrated to be involved in many pivotal processes of the innate and adaptive immune systems [10], [11]. To explore the role of IL-23 in pathogenesis of hepatitis B, we first assessed the expression of IL-23 in liver tissues from healthy individuals and patients with CHB or ACLF. Results showed that IL-23 transcripts and protein were strongly expressed in liver tissues of ACLF and CHB patients, as compared to that detected in tissues from healthy controls. Moreover, the liver tissues of ACLF patients exhibited a much higher expression level of IL-23 over that in CHB livers (**Figure 1A and B**). The high IL-23 expression in the livers of CHB and ACLF patients was further confirmed by *in situ* immunohistochemistry assay. We found that the frequency of IL-23^+^ cells was significantly increased in liver tissues of infected patients, as compared to healthy controls, and those cells were mainly located in the portal tract (**Figure 1C**). The significant difference of IL-23 expression among ACLF, CHB and healthy controls suggests an association between IL-23 and liver damage in HBV infection.

**Figure 2:**
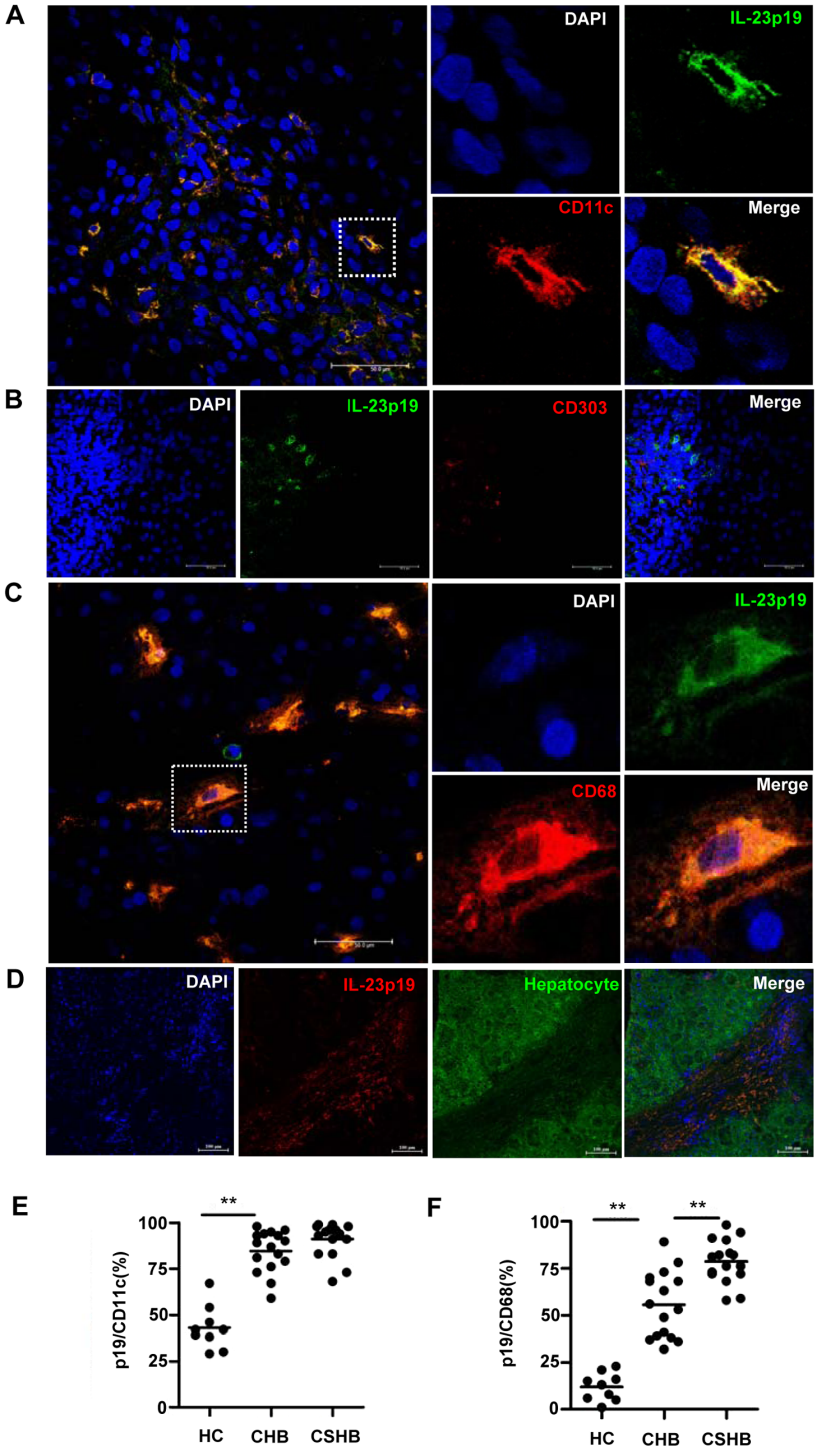
IL-23 is mainly derived from APCs in hepatitis B liver tissue. Co-localization (yellow) of IL-23 (p19, green) with mDC (CD11c, red) (**A**), pDC (CD303, red) (**B**), macrophage (CD68, red) (**C**), or IL-23 (p19, red) with hepatocyte (hepatocyte, green) (**D**) in CHB patients' liver tissues was detected by immunofluorescence staining. The right panels are enlarged images of the area in the left panel that is demarcated by a white dashed-line (**A** and **C**). (**E** and **F**), The frequencies of intrahepatic IL-23-positive mDCs or macrophages in the livers of patients with hepatitis B and healthy controls are shown. A total of 118 CD11c^+^ and 89 CD68^+^ cells were counted in 10 random fields (magnification 400×, 0.066 mm^2^) per section using a digital camera. Error bars indicate SD. **P<0.01.

To further confirm the source cell type of IL-23, we performed immunofluorescence staining on liver tissues from patients with hepatitis B. Confocal microscopy of liver tissues showed that IL-23p19, the specific subunit of IL-23 heterodimers, co-localized with the CD11c mDC marker, but not with the CD303 pDC marker (**Figure 2A and 2B**). Besides DCs, macrophages are another important innate immune cell and APC type. Therefore, we investigated whether liver macrophages were also responsible for IL-23 production upon HBV infection. Confocal microscopy assay showed that most of the macrophages but not the hepatocytes in hepatitis B patients secrete IL-23 (**Figure 2C and 2D**).

Immunofluorescence assay of liver biopsy samples from healthy controls showed IL-23 staining in less than 50% of mDCs, while those from hepatitis B patients showed staining in over 80% of mDCs (**Figure 2E**). Similarly, only about 10% of macrophages in the samples from healthy controls were IL-23-positive, while over 60% of macrophages in the hepatitis B samples expressed IL-23 (**Figure 2F**).

These results indicate that the elevated IL-23 expression in livers of hepatitis B patients is mainly derived from APCs, including mDCs and macrophages, and suggests that these cells are likely involved in the immunological responses to HBV.

### IL-23R expression is enhanced markedly and correlated with the IL-23 level in liver tissues of hepatitis B patients

**Figure 3:**
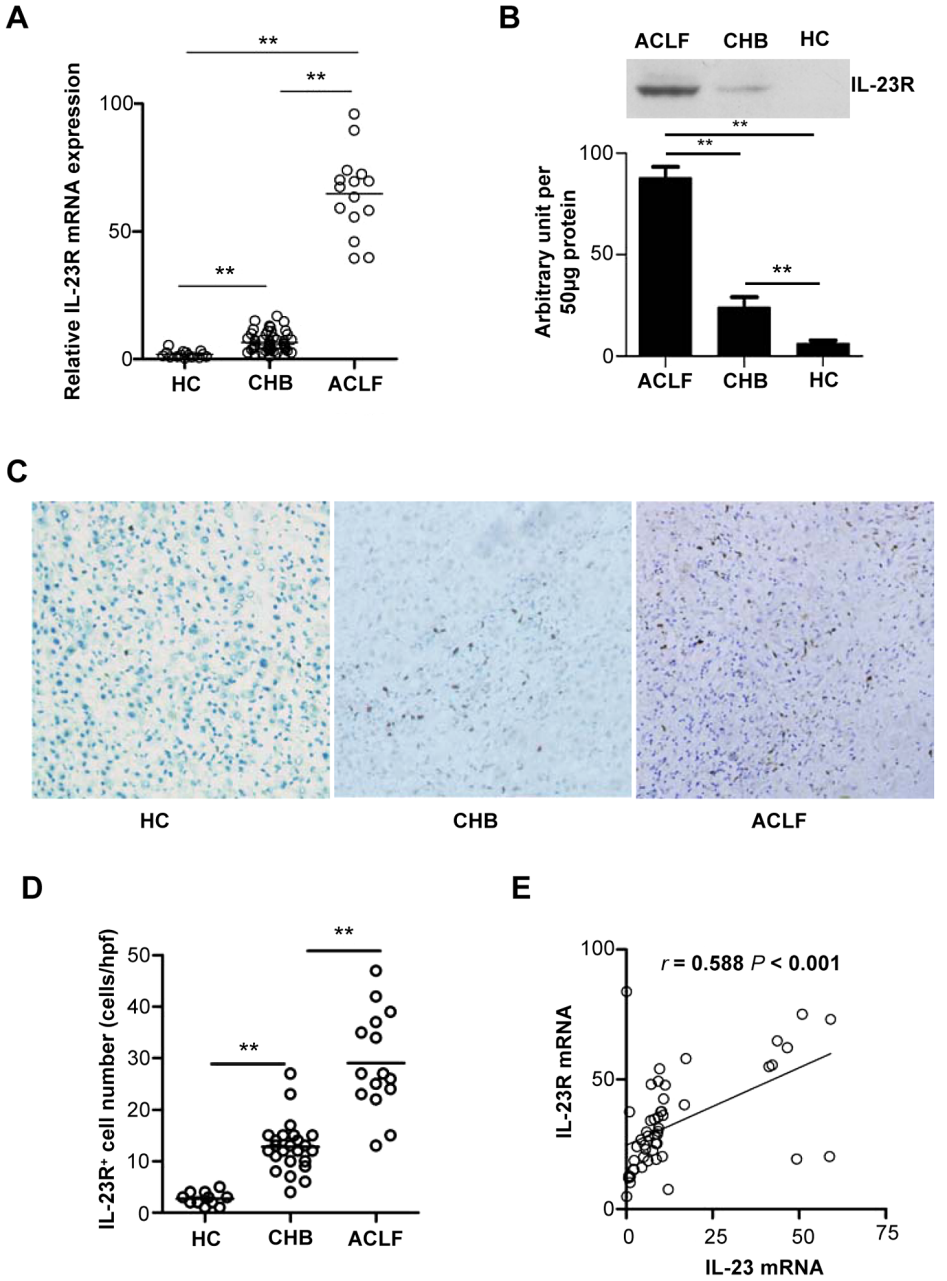
IL-23R expression is enhanced markedly and correlated with IL-23 level in liver tissues of CHB and ACLF patients. (**A**) Relative mRNA expression of IL-23R in liver tissue of various groups was analyzed by qRT-PCR. (**B**) Protein expression of IL-23R was analyzed by Western blot. Error bars indicate SD. **P<0.01. (**C**) *In situ* expression of IL-23R in liver tissues was detected by immunohistochemical staining (magnification 100×). (**D**) The frequencies of intrahepatic IL-23R positive cells in patients with hepatitis B and health controls are shown. Error bars indicate SD. **P<0.01; HPF, high power fields. (**E**) The correlation was analyzed between the expression of IL-23 and IL-23R in HBV infected liver tissue. *r* is the correlation coefficient and *P*-value is shown.

As a functional cytokine, IL-23 functions only if it binds to its cognate receptor, IL-23R, which is found to be mainly expressed by activated T cells [10]. To investigate the potential effects of IL-23 on hepatitis B, we evaluated the expression of IL-23R in HBV infected liver tissues. Results showed that the mRNA and protein expression levels of IL-23R in liver tissue infected with hepatitis B were significantly higher than in tissues from healthy controls (**Figure 3A and 3B**). Most of the IL-23R^+^ cells were located in the portal tract, and the frequency of IL-23R^+^ cells in liver tissue of patients with hepatitis B was remarkably higher than in liver from healthy controls (**Figure 3C and 3D**). Furthermore, the expression of IL-23R was significantly correlated with IL-23 in liver tissue with hepatitis B (*P*<0.001, **Figure 3E**).

### IL-17 in liver tissue with hepatitis B is mainly derived from CD4^+^IL-23R^+^ T (Th17) cells and significantly relates with IL-23/IL-23R expression

**Figure 4:**
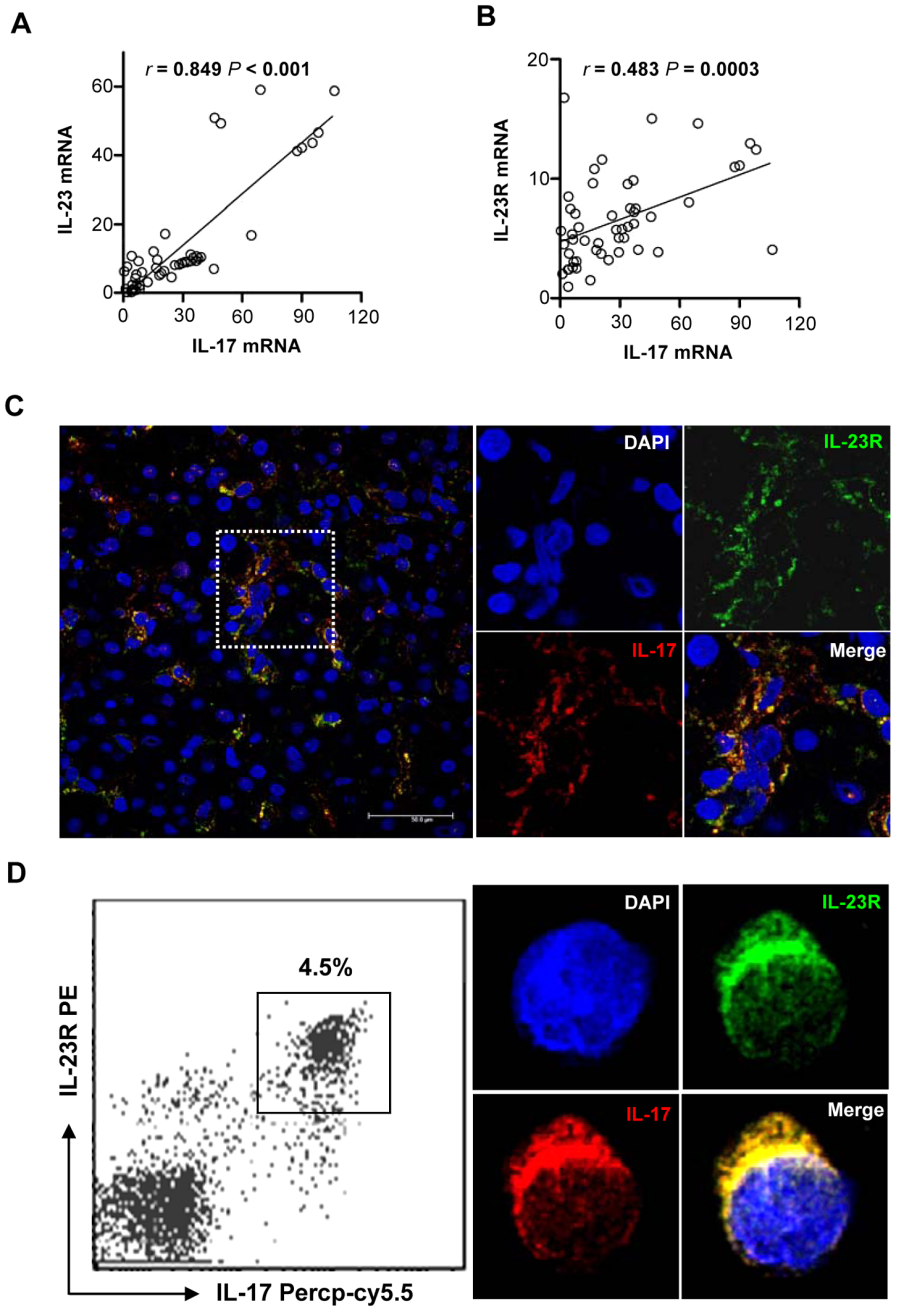
Hepatic IL-23/IL-23R is closely correlated with elevated IL-17, and IL-23R^+^ T cells are the main source of IL-17 in liver of CHB and ACLF patients. Relative mRNA expressions of IL-23, IL-23R and IL-17 in liver tissues infected by HBV were assayed by qPCR, and then Spearman's rank correlation was used to determine the correlation between relative mRNA expression of (**A**) IL-23 or (**B**) IL-23R and IL-17 in 51 CHB patients. Co-localization of IL-17 (red) with IL-23R (green) (**C**) in CHB patients' liver tissues was examined by fluorescence microscope. The right panels are enlarged images of the area in the left panel demarcated with the white dashed-line box. (**D**) Co-localization of IL-17 and IL-23R in PBMCs from hepatitis B patients is shown. PBMCs from patients with CHB were stimulated by PMA and ionomycin for 4 h in the presence of GolgiStop. A moiety of the stimulated PBMCs was measured by FCM to determine the frequency of IL-17^+^23R^+^ T cells in the total CD4^+^ T cells, with gating on CD3^+^CD8^−^ T cells. Co-localization of IL-17 (red) and IL-23R (green) in the smear of stimulated PBMCs was examined by confocal microscopy. The data shown are representative from one of the five CHB patients.

Since IL-23/IL-23R expression was significantly elevated in livers of patients infected by HBV (**Figures 1** **–** **3**) and IL-23 is essential for the functional maintenance of Th17 cells [15], we anticipated that IL-17 expression might be enhanced in HBV infected liver tissues, as a result of the elevated IL-23 expression. Indeed, we found that IL-17 expression was significantly elevated in the livers of CHB and ACLF patients, as compared to that in healthy controls (**Figure S1A–C**). Furthermore, IL-17 expression in liver tissues of ACLF patients was higher than that in CHB patients, which was in agreement with a previous report of elevated Th17 cell frequency in hepatitis B patients [22]. We also observed that the IL-17 expression levels in liver were much higher than that in PBMCs from the same hepatitis B patients (**Figure S1D**). Further statistical analyses confirmed a significant positive correlation between IL-23/IL-23R and IL-17 expression levels in liver biopsies from the patients with CHB (*P*<0.001; **Figure 4A and 4B**).

To explore the role of IL-23 on IL-17 production in hepatitis B pathogenesis, we investigated which cell type(s) adopted IL-23 signals to produce the pathogenic IL-17 by detecting the expression of IL-23R in IL-17^+^ cells in liver tissue. Confocal microscopy revealed that IL-17 was almost completely co-localized with IL-23R in hepatitis B-infected liver tissues (**Figure 4C**). Moreover, although not all CD4^+^ T cells expressed IL-17, the IL-17 was mainly expressed in CD4^+^ T cells and only rarely expressed in γδT cells or neutrophils (**Figure S2**). When determining the expression of IL-23R in PBMCs of patients with CHB by flow cytometry (FCM) analysis, we also found that most IL-23R^+^ cells were IL-17^+^CD4^+^ T cells (**Figure 4D**, left panel), which indicated that the circulating Th17 cells expressed a high level of IL-23R under the hepatitis B condition. Confocal microscopy results further confirmed the co-expression of IL-17 and IL-23R in PBMCs (**Figure 4D**, other panels). Together, these results indicate that IL-23 might stimulate CD4^+^IL-23R^+^ T (Th17) cells within HBV-infected livers and PBMCs to produce the pathogenic IL-17.

### Intrahepatic IL-23/IL-17 expression is correlated with the phenotypes of liver damage in HBV infected patients

**Figure 5:**
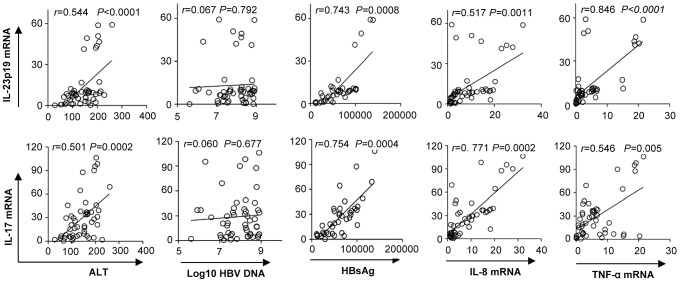
*In situ* infiltration of IL-23 and IL-17 is significantly correlated with liver injury. Correlations were analyzed between IL-23 or IL-17 and either plasma log10 HBV loads (copies/mL), serum ALT levels (U/L), HBsAg (IU/mL; concentration above 10^4^), IL-8, or TNF-α. Solid line indicates a linear growth trend. *r* is the correlation coefficient. *P*-values are shown.

To further address the role of IL-23/IL-17 pathway in pathogenesis of HBV infection, we analyzed the relationship between the expression of IL-23/IL-17 and clinical phenotypes of CHB patients. Results in **Figure 5** showed that the expression of IL-23 and IL-17 in livers of CHB patients was significantly correlated with the serum alanine aminotransferase (ALT) level and the proinflammatory cytokines, including tumor necrosis factor (TNF)-α and IL-8, but not with the plasma HBV load. Interestingly, we found that there was a remarkable correlation between the expressions of IL-23/IL-17 and the level of serum HBsAg, when the HBsAg concentration was above 10^4^ IU/mL, suggesting that only a high enough level of HBsAg could efficiently induce the production of IL-23/IL-17.

### HBsAg induces IL-23 production by APCs in a MR-dependent manner and IL-10 inhibits the IL-23 production from DCs

**Figure 6:**
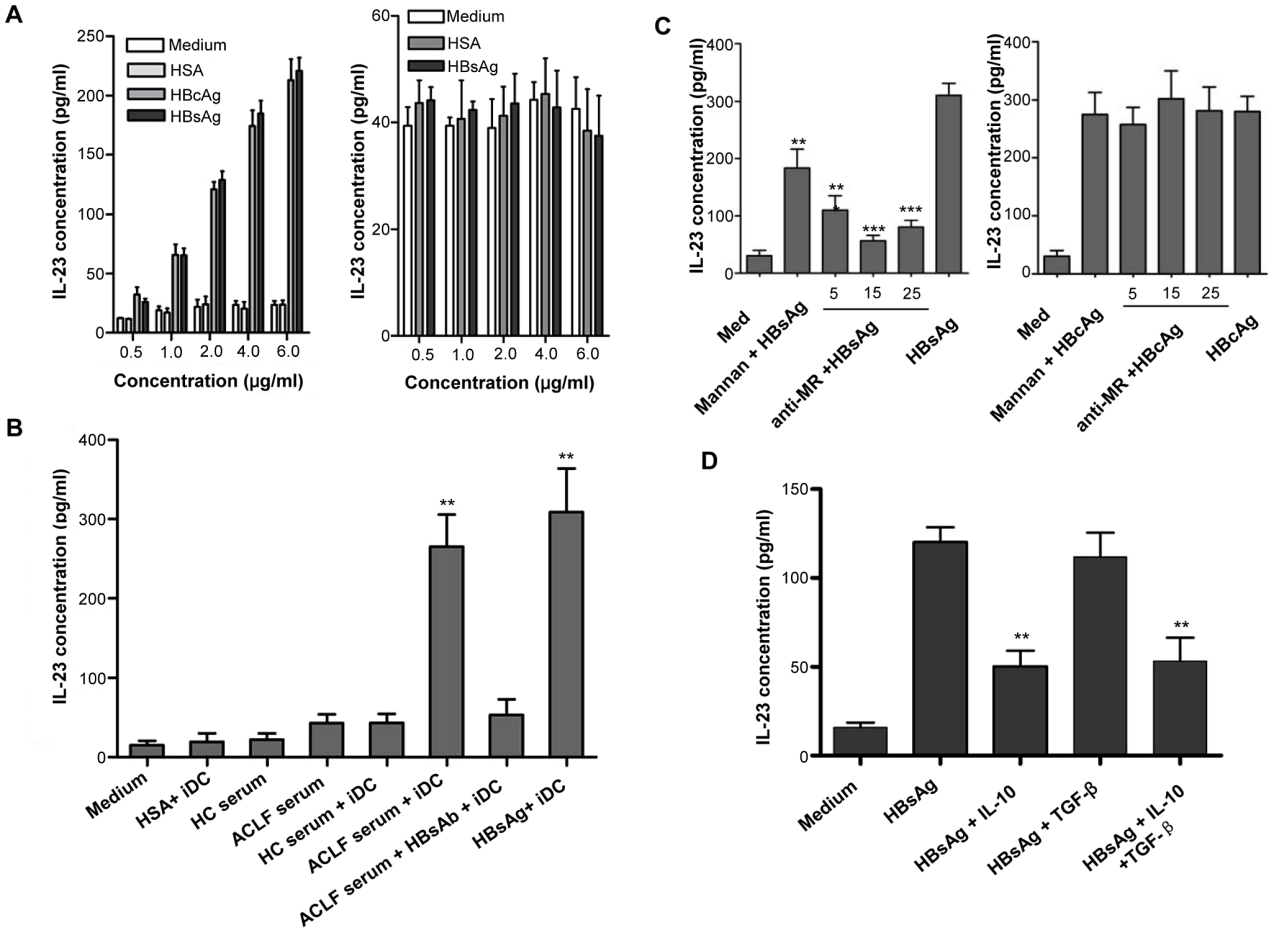
HBsAg induces DCs and macrophages to produce IL-23 in a MR-dependent manner. (**A**) Monocytes were isolated from PBMCs of healthy blood donors and mDCs were induced by culturing in the presence of GM-CSF and recombinant human IL-4 for five days. Then, the mDCs were stimulated by various doses of HBsAg or HBcAg. ELISA was used to detect the concentrations of IL-23 and IL-12 in the supernatants. (**B**) mDCs were stimulated by 50 µL serum from healthy controls (HC) or 50 µL serum from ACLF patients (HBsAg concentration: 79876.4 IU/mL, equivalent to 8 µg/mL) in a 200 µL culture system. For the blocking group, the ACLF serum had been pretreated with HBsAg antibody at a final concentration of 50 µg/mL for 30 min. The concentrations of IL-23 in the supernatants were detected by ELISA. The data represent one of three independent experiments with similar results. Error bars indicate SD. **P<0.01 vs. all other groups. (**C**) mDCs preincubated with mannan (100 µg/mL) or various doses of neutralizing anti-MR antibody for 30 min were stimulated by HBsAg or HBcAg (2 µg/mL, equal to that of HBsAg). Controls were preincubated with medium alone. ELISA was used to detect the concentration of secreted IL-23 in the supernatants. The data represent one of three independent experiments with similar results. Error bars indicate SD. **P<0.01; ***P<0.001 vs. the HBsAg group. (**D**) Monocytes were isolated from PBMCs of healthy blood donors and mDCs were induced by culturing for five days in the presence of GM-CSF (50 ng/mL) and recombinant human IL-4 (5 ng/mL). Then, mDCs were stimulated by HBsAg (2 µg/mL) alone or with recombinant human IL-10 (10 ng/mL) [27] or with TGF-β (2 ng/mL) [55], [56] for 48 hours. The supernatant was collected and IL-23 level was detected by ELISA. The data represent one of three independent experiments with similar results. Error bars indicate SD. **P<0.01.

HBV-infected patients display large amounts of HBV particles and viral proteins in their circulation, with HBsAg being predominant. As such, there is substantial opportunity for interaction between HBV antigens and APCs *in vivo*. Since IL-23 appeared to mainly be derived from APCs and IL-23 expression was closely correlated with plasma HBsAg level (**Figure 5**), we investigated whether the main HBV-derived antigen, HBsAg, could directly prime APCs, such as DCs and macrophages, to secrete IL-23 *in vitro*. In addition, another important HBV antigen, HBcAg, was also investigated in parallel. Results showed that treatment of human monocyte-derived DCs with recombinant HBsAg or HBcAg led to a dose-dependent increase in IL-23, but had little effect on IL-12 expression, as compared with the medium-only control and the human serum albumin (HSA) negative control (**Figure 6A**). Similar results were obtained in monocyte-derived macrophages upon stimulation by HBsAg or HBcAg (data not shown). As the HSA and HBV antigens were prepared by the same protocol using a mammalian CHO-cell system, it was unlikely that contamination of lipopolysaccharide endotoxin or differential arrays of other Toll-like receptor (TLR) ligands would significantly affect the experiment. To further explore the mechanisms of HBsAg-stimulated IL-23 production from mDCs of patients with hepatitis B, patients' sera with high concentrations of HBsAg were added into the culture system. As shown in **Figure 6B**, the sera from patients with ACLF significantly induced IL-23 production from mDCs, but this effect was sharply reversed by addition of HBsAg antibody (HBsAb) prepared from the anti-sera of healthy volunteers who received inoculation of preventive HBsAg vaccine and were HBsAb-positive. Similar results were obtained *in vitro* with macrophages stimulated by HBsAg or patients' sera (**Figure S3**).

Since DCs have been shown to uptake HBsAg through their surface mannose receptors [23], we investigated whether interaction between the glycoprotein HBsAg and the MR is responsible for the production of IL-23 from DCs. To this end, DCs were treated with mannan (as a competitive inhibitor [23]) or increasing amounts of neutralizing anti-MR antibodies to assess the effects HBsAg on MR-blocked DCs. As shown in **Figure 6C**, both mannan and MR-blocking antibody effectively decreased the HBsAg-induced IL-23 production from DCs, suggesting that HBsAg could induce IL-23 expression by binding to and signaling through the MR on DCs. However, IL-23 production from DCs induced by HBcAg was unaffected by mannan or the neutralizing antibody targeting the MR (**Figure 6C**), indicating that HBcAg stimulated the DCs in a MR-independent manner. Similar results were observed when macrophages were used in this assay, which was expected since macrophages also express MR [24] (data not shown).

In our previous studies, we found that the frequencies of both Treg and Th17 cells were increased in hepatitis B patients [12], [25]. However, Th17 cells might be inhibited by the functional cytokines of Treg cells, such as IL-10, which has been demonstrated to suppress the production of Th17 cytokines by down-regulating the RORγt expression [26], [27]. Therefore, in this study, we further investigated whether the critical cytokines of Treg cells (i.e. IL-10 and TGF-β) could influence Th17 cells through affecting the IL-23 production from mDCs. Results showed that the exogenous IL-10 protein significantly inhibited the HBsAg-induced IL-23 production by mDCs; however, TGF-β addition did not influence the IL-23 production from HBsAg-stimulated mDCs (**Figure 6D**).

### Response of DCs and macrophages to HBsAg via endocytosis

**Figure 7:**
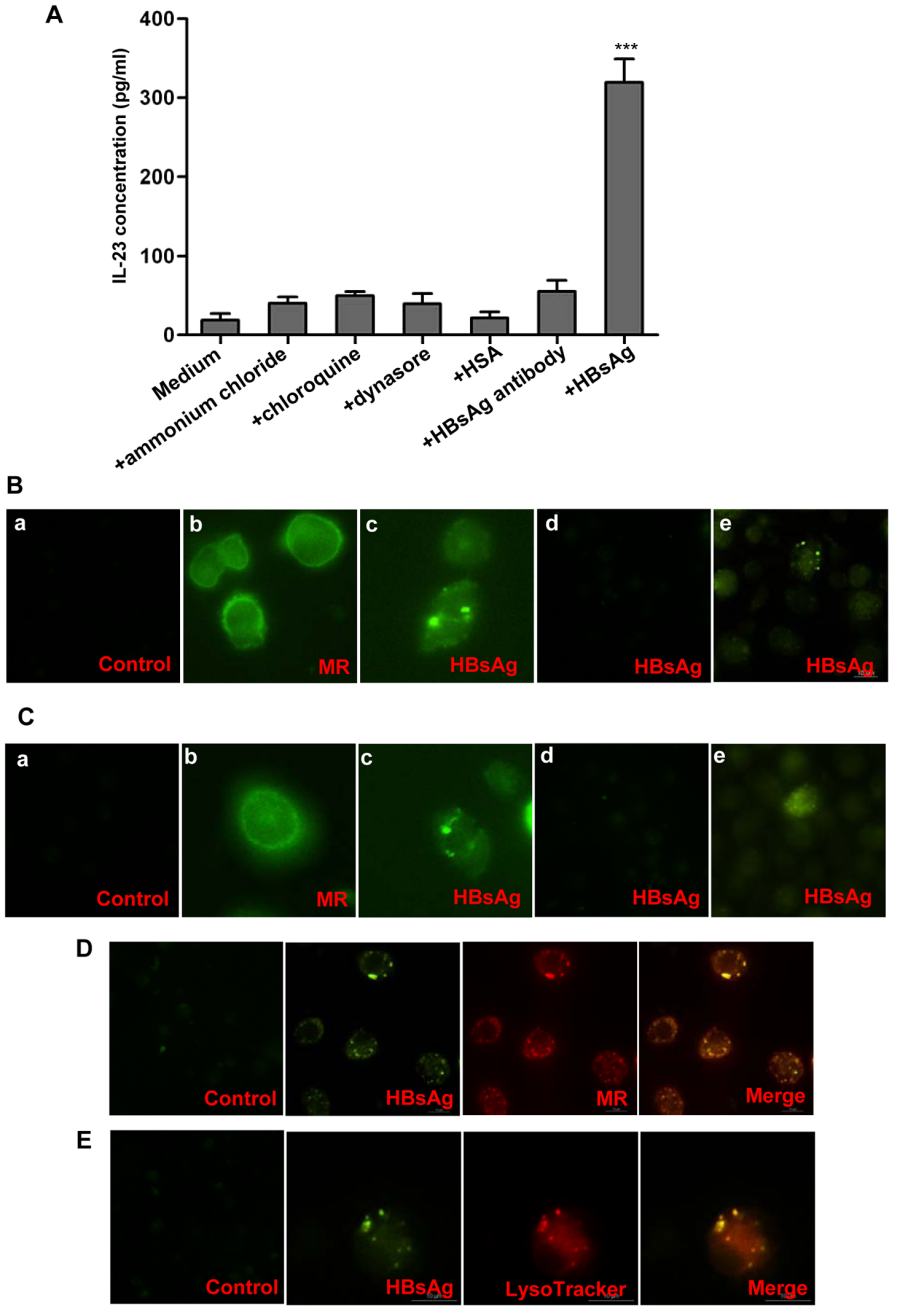
IL-23 production from DCs and macrophages stimulated by HBsAg is dependent upon endocytosis. (**A**) Monocytes were isolated from PBMCs of healthy blood donors and mDCs were induced by culturing in the presence of GM-CSF (50 ng/mL) and recombinant human IL-4 (5 ng/mL) for five days. Then, the mDCs were stimulated by HBsAg with or without ammonium chloride (10 µM), chloroquine (10 mM), Dynasore (80 µM), or HBsAg antibody (50 µg/mL) for 40 hours. ELISA was used to detect the concentration of IL-23 in the supernatants. The data represent one of three independent experiments with similar results. Error bars indicate SD. ***P<0.001 vs. all other groups. DCs (**B**) and macrophages (**C**) were obtained from PBMCs for different stimulations and stainings. (**a**) Cells were stimulated with HBsAg (2 µg/mL) for 45 min and stained by isotype IgG (FITC). (**b**) Cells were directly stained with anti-MR mAb (FITC) without any stimulations. (**c**) Cells were stimulated with HBsAg (2 µg/mL) for 45 min and stained by anti-HBsAg mAb (FITC). (**d**) Cells were pretreated with MR-blocking antibody (10 µg/mL) for 30 min before the cells were stimulated by HBsAg (2 µg/mL) for 45 min and stained with anti-HBsAg (FITC). (**e**) Cells were pretreated with chloroquine (10 mM) for 10 min before stimulation by HBsAg (2 µg/mL) for 45 min and staining with anti-HBsAg (FITC). mDCs were stimulated by HBsAg (2 µg/mL) for 45 minutes, and the respective co-localizations of HBsAg with MR (**D**) or endosomal marker LysoTracker (**E**) were detected by confocal microscopy. (magnification 400×).

To further dissect the mechanisms responsible for IL-23 production, we investigated whether the IL-23 production from DCs was dependent upon endocytosis or endosome acidification. The HBsAg-stimulated high-level secretion of IL-23 by DCs was found to be significantly blocked in the presence of ammonium chloride, chloroquine or Dynasore, similar to the results obtained with HBsAb blocking (**Figure 7A**). Moreover, a similar trend was observed in macrophages (data not shown). Immunofluorescence assay showed that the cell surface of both DCs and macrophages bound much of the FITC-labeled anti-MR antibody, indicating the rich expression of MR on these cells (**Figure 7B(b)**). When HBsAgs were incubated with DCs, they appeared to be internalized, as evidenced by the FITC-anti-HBsAg-mAb staining pattern (**Figure 7B(c)**). However, this internalization was dependent on endocytosis and was MR-mediated, and was almost completely blocked by pretreatment with chloroquine or MR-blocking antibody (**Figure 7B(d**–**e)**). A similar trend was observed with macrophages (**Figure 7C**). To verify these results, we further examined the co-localization of MR/HBsAg, and HBsAg/endosomal marker LysoTracker by confocal microscopy. We found that HBsAg was almost completely co-localized with MR or LysoTracker (**Figure 7D and 7E**), indicating that HBsAg was taken up by mDCs through binding to MR and subsequent endocytosis.

To confirm whether HBcAg-stimulated IL-23 production from mDCs was an endocytosis-dependent process, mDCs were stimulated by HBcAg alone or in the presence of ammonium chloride, chloroquine, Dynasore, or HBcAg-blocking antibody for 40 hours. Effects on IL-23 production by the mDCs were examined by FACS, and the IL-23 level was found to be significantly decreased by the HBcAg-blocking antibody, but only minimally decreased by ammonium chloride, chloroquine and Dynasore (**Figure S4**). These results suggest that HBcAg-stimulated IL-23 production in mDCs occurs through a certain yet unknown pathway, other than endocytosis. We also observed similar results with HBcAg-stimulated macrophages (data not shown).

As shown in **Figure 7**, IL-23 production by DCs appeared to be significantly blocked by ammonium chloride, chloroquine, and Dynasor. However, the absence of IL-23 production could merely reflect poor viability of DCs since these reagents are toxic under some culture conditions [28]–[30]. To confirm that the DCs remain fully functional in our experimental system, we examined DCs responsiveness to LPS stimulation in the presence of each of these reagents because LPS activates host cells by TLR4 signaling, which does not require endocytosis or endosomal acidification. After 12 hours of stimulation with LPS in the presence of chloroquine, the expression patterns of co-stimulatory molecules, including CD40, CD80/86, CD274 and HLA-DR, on DCs were nearly identical to those on DCs stimulated with LPS in the absence of chloroquine (**Figure S5**). In addition, similar results were achieved with stimulation in the presence of ammonium chloride or Dynasor (data not shown).

### IL-23 is indispensable for HBV antigen-stimulated IL-17 production, and hepatic stellate cells (HSCs) and mDCs are the potential target cells of IL-17

The significantly positive correlations that were observed between IL-23/IL-23R and IL-17 expression in patients with hepatitis B led us to investigate whether IL-23 is indispensable for HBV antigen-stimulated IL-17 production. Naïve CD4^+^ T cells that were co-cultured with HBsAg or HBcAg pretreated DCs produced a significant amount of IL-17 and showed a markedly higher frequency of IL-17^+^ T cells than controls. Addition of recombinant IL-23 further enhanced these effects, while addition of IL-23-blocking antibody dramatically decreased these effects (**Figure S6**). Together, these data suggest that IL-23 derived from HBV antigen-stimulated mDCs can effectively polarize naïve CD4^+^ T cells toward the IL-17-producing Th17 phenotype. This finding might at least partially reflect the *in vivo* effects of HBV infection on mDCs and the subsequent effects on Th17 cells.

It is well known that cytokines initiate downstream signaling pathway activities by binding to specific receptors expressed on the target cells, thus IL-17 should also bind to its cognate receptor to mediate liver injury during HBV infection. IL-17 receptors are reported to be expressed on several kinds of cells including epithelial cells, fibroblasts, B and T lymphocytes, myelomonocytic cells, and marrow stromal cells [31]. However, the IL-17R expression pattern in hepatitis B liver should be clarified specifically. To this end, we first examined the IL-17R expression in the livers of CHB patients by Western blot assay and *in situ* immunostaining. Results demonstrated that IL-17R expression was appreciably higher in patients with hepatitis B infection and the expression was mainly in the fibrotic septa (**Figure S7A** and **B**). The localization of positively stained cells, and the essential role of HSCs in hepatic fibrosis prompted us to investigate whether the cells observed as IL-17R^+^ were in fact HSCs. By double-fluorescence staining, we confirmed that the IL-17R was indeed expressed on HSCs (**Figure S7C**). We further found that a few mDCs also expressed IL-17R (**Figure S7D**). These results indicate that HSCs and mDCs might be the potential target cells of IL-17 in the liver of hepatitis B patients.

## Discussion

Proper inflammatory response is crucial to the positive outcome of patients infected by hepatitis B virus [32], [33]. The host IL-23 signaling pathway is critical to the immune response and is the target of manipulation by pathogens seeking to establish infection [34], [35]. Here, we describe our investigations into the role of intrahepatic IL-23/IL-23R signaling during development of lethal or chronic hepatitis. We found that the IL-23/IL-23R pathway was strongly activated in liver tissues from HBV infected human patients, as compared with those from healthy controls. Moreover, we determined that in these patients IL-23 was principally derived from the intrahepatic myeloid DC population and macrophages. When stimulated by HBsAg and HBcAg *in vitro*, monocyte-derived DCs and macrophages produced large amounts of IL-23. The effect for HBsAg was dependent on the availability and function of the surface MR, while the mechanisms underlying the HBcAg-induced effect remain unclear. After binding to MRs, HBsAg entered the cells via endocytosis, which subsequently induced the polarization of Th17 cells from naïve CD4^+^ T cells and IL-17 production. In addition, IL-17R expression was mainly detected on HSCs and a few mDCs in liver tissue, suggesting that these cells may be potential targets of IL-17 in hepatitis B patients.

IL-23 is a heterodimeric cytokine composed of an IL-12 shared p40 subunit and a unique p19 subunit [9], [10], and it is primarily produced by APCs upon encounter with pathogen associated molecular patterns (widely known as PAMPs) [10], [36]. In HBV infection patients, we found that IL-23 expression was largely elevated, as compared to that detected in the healthy controls, which agreed with the previous observations reported by Xia et al. in hepatitis B patients [5]. In the current study, the concentration of HBsAg in serum was found to be positively correlated with IL-23 expression for patients with hepatitis B. Confocal fluorescence microscopy further confirmed that mDCs and macrophages, and not pDCs or hepatocytes, were the main source cell types for IL-23 production. Surprisingly, Xia et al. had demonstrated that the hepatocytes were the main cell types for IL-23 production and that the HBx antigen acted as the active transcription factor for the IL-23 gene in liver cells [5]. These apparently incongruous findings may simply reflect the different experimental methods used in each study; for example, we used a fluorescence confocal microscopy strategy that may more directly show the source cells than the routine immunohistochemical analysis used by Xia et al. Furthermore, Xia et al. used an HBx-overexpressing plasmid-transformed liver cell line that may not reflect the real intrahepatic condition in hepatitis B patients as closely as the freshly isolated human cells or human liver tissues used in our study.

It has been shown that the frequencies of both Treg and Th17 cells are higher in hepatitis B patients than in healthy controls [12], [25]. In addition, Treg cells have also been shown to inhibit the function of Th17 cells by secreting key inhibitory cytokines, such as IL-10, which suppress the function of Th17 cells by negatively regulating the production of Th17-related proinflammatory cytokines by down-regulating RORγt expression in PBMCs [26], [27]. Therefore, in this study, we further investigated whether IL-10 and another key cytokine of Treg cells, TGF-β, could exert negative regulatory effects on Th17 cells by suppressing the production of IL-23 in mDCs, which is known to be a critical step in the differentiation and maintenance of Th17 cells [14], [15]. In an *in vitro* system, we found that the exogenous IL-10 protein significantly inhibited the HBsAg-induced IL-23 production by mDCs; however, TGF-β addition did not influence the HBsAg-stimulated IL-23 production from mDCs. Recent studies have shown that IL-10 levels in hepatitis B patients are significantly higher than in non-infected individuals [37], [38]; nevertheless, no significant differences were observed between chronic hepatitis B patients and healthy controls for the levels of TGF-β [38]. Notwithstanding the increased IL-10 level in hepatitis B patients, we observed high-level IL-23 expression and Th17 frequency in these patients, suggesting that other mechanisms likely exist to support Th17 function by antagonizing the suppressive effects of IL-10 during HBV infection *in vivo*.

The primary pathogenic mechanism of HBV involves direct infection of hepatocytes. However, it is possible that HBV and its antigens may also be recognized and taken up by host immune cells, especially DCs and macrophages, which then provoke the anti-HBV immune responses. It is known that DCs can recognize HBV antigens by at least three different mechanisms [39]. First, HBV DNA binding to TLR9 on DCs activates the NF-κB signaling pathway that results in secretion of type I IFN and inflammatory cytokines, and induces DC maturation and the adaptive immune response [39], [40]. Second, unlike HBc/HBeAg which seem to be recognized only by the B cells [41], HBsAg appears to be able to be recognized by APCs, such DCs. While several putative binding factors have been described for this interaction, including asialoglycoprotein receptor and mannose-binding lectin, their exact role in HBV attachment to and uptake by DCs remains unclear [42]. In one of the more recent studies, the MR on intrahepatic DCs was shown to functionally interact with HBsAg [43]. Considering that macrophages also express MRs [24], it is probable that these cells will be able to uptake HBsAg in a similar manner. In this study, we observed that blocking MR with MR-neutralizing antibody or blocking HBsAg with antibody obtained from healthy volunteers, who had received inoculation of preventive HBsAg vaccine and were HBsAb-positive, significantly suppressed HBsAg (HBsAg protein or ACLF serum)-induced IL-23 production by mDCs and macrophages. We further verified that the MR-mediated IL-23 production from mDCs and macrophages was dependent on an endocytosis mechanism because such stimulations were significantly decrease in the presence of inhibitors of endosomal acidification, such as ammonium chloride and chloroquine, or of the cell permeable dynamin inhibitor Dynasore that blocks cell endocytosis. Immunofluorescence assay further clearly showed that the cell surface of both DCs and macrophages express rich MR protein. HBsAgs could be efficiently internalized by mDCs and macrophages in an endocytosis and such internalization was MR-mediated because the internalization was almost completely blocked by pretreatment with chloroquine or MR-blocking antibody. Furthermore, we found that HBsAg almost completely co-localized with MR or the endosomal marker LysoTracker. This finding indicates that MR on DCs and macrophages not only acts as an endocytic receptor of HBsAg but also mediates an intracellular signaling response that is triggered upon encounter with HBV-related antigens [44].

Studies of fungal pathogens have shown that MR can induce production of a number of cytokines, including IL-8, IL-10, IL-17, TNF-α and MCP-1, as well as inhibit that of others, such as IL-12 and TNF-α [45], [46]. However, how the downstream molecular signaling mechanisms of MR have not yet been defined because the MR cytoplasmic tail is devoid of any classical signaling motifs [45]. Accordingly, it has been theorized that MR requires functional interactions with other receptors, such as TLR2 and FcRγ, to trigger any signaling cascades [45], [46]. As such, the role of MR in host immune response to a pathogenic infection represents an additional level of complexity to studies attempting to understand the cellular activation process.

Moreover, we found that not only synthetically engineered HBsAg protein but also the ACLF patients' sera containing large amounts of HBsAg were able to directly induce mDCs and macrophages to secrete IL-23 protein in a dose-dependant manner *in vitro*. All these effects were reversed upon exposure to the antibody obtained from healthy volunteers who had received inoculation of the preventive HBsAg vaccine and were HBsAb-positive. These data demonstrated the specific effects of HBsAg on mDCs and macrophages, and, more importantly, provided clinical evidence of HBsAg function as a stimulator of IL-23 production from host cells. Although no definitive receptors for HBcAg have been reported to date, according to our data, HBcAg can stimulate DCs and macrophages to produce IL-23 in a MR-independent manner. The underlying mechanisms remain undefined, but may include: 1) binding to currently unknown HBcAg receptor(s) and triggering a downstream signaling pathway; 2) phagocytosis by phagocytes such as mDCs and macrophages, and thereby initiating intracellular signal transmission. Future studies should investigate these hypotheses.

In addition, our results in this study, along with the observations from Zhang *et al.* [4], verified that both Th17 cells and IL-17 are increased and correlated with the clinical phenotypes of hepatitis B patients. However, in order to understand the functions of the IL-23/IL-23R axis related to liver damage caused by HBV infection, the interplay between elevated IL-23/IL-23R and IL-17 in hepatitis B patients has to be clarified. It was found that mice deficient in IL-23p19 were resistant to experimental allergic encephalitis, and failed to produce IL-17 [47]. Meanwhile, Aggarwal *et al.* found that IL-23 was able to stimulate secretion of IL-17 from memory T cells *in vitro* [48]. It has also been demonstrated that IL-23 signaling is required for the terminal differentiation of activated T cells into functional effector Th17 cells in mice [15]. In humans, the factors involved in Th17 cell development and regulation are more complicated; however, among the potential factors, the critical role of IL-23 for the function of Th17 cells has also been observed in most studies (reviewed in reference [49]). In the present study, we found that IL-23R in HBV-infected liver tissue was mainly expressed on IL-17^+^ cells, indicating the *in situ* IL-17 production being dependent upon the IL-23/IL-23R axis. Meanwhile, in the co-culture experiments, IL-23 derived from mDCs and macrophages was able to stimulate the secretion of IL-17 from CD4^+^ T cells in the presence of HBV antigen; however, IL-23-blocking antibody significantly decreased the production of IL-17. Thus, these results firstly demonstrated that the IL-23/IL-23R axis was indispensable for the elevated IL-17 production that occurs in HBV infection cases. However, we cannot exclude potential roles for other factors, such as IL-1β and IL-6 in Th17 cell function because we found that blocking the IL-23/IL-23R axis did not diminish all of the IL-17 expression or completely eliminate Th17 cells from the system.

IL-17 is considered the principal mediator of Th17 cell functions. In several experimental disease models, IL-17 binding to the IL-17 receptor on target cells has been shown to induce the expression and secretion of proinflammatory factors, such as IL-6 and IL-8, which in turn mediate the inflammatory response [31], [50]. Zhang *et al.* reported that IL-17R was uniquely expressed on peripheral monocytes and mDCs in CHB patients [4]. *In vitro* analysis in that study also revealed that IL-17 can activate mDCs and monocytes and enhance their capacity to produce proinflammatory cytokines. However, the authors did not detect IL-17R expression on the mDCs and monocytes from the liver tissue of CHB patients. This negative finding might simply be due to the technical difficulty associated with detecting IL-17R positive cells by using a chromogenic immunohistochemisty method. Even though we used the much more sensitive immunofluorescence method, we could only detect IL-17R expression on a few mDCs from the liver tissue of CHB patients. Nevertheless, we detected a substantial amount of IL-17R positive cells in the liver tissue, mainly within the fibrotic septa area, and these cells were subsequently identified as HSCs. We further verified that a few mDCs in the liver tissue of hepatitis B patients could also express IL-17R, suggesting that both HSCs and mDCs might be target cells of IL-17 and involved in the pathogenesis of hepatitis B disease.

In conclusion, the findings from this study demonstrate that the IL-23/IL-23R axis is highly activated and closely correlated with Th17 cells, liver damage and clinical phenotypes of hepatitis B patients. The HBsAg binds to the MRs on mDCs and macrophages, enters cells by endocytosis, and finally efficiently stimulates those mDCs and macrophages to produce IL-23. However, IL-10, and not TGF-β, was able to decrease IL-23 production from mDCs in an *in vitro* system. In contrast, HBcAg could stimulate mDCs to secrete IL-23 via a yet unknown mechanism that functions in an MR- and endocytosis-independent manner. IL-23 induction of naïve CD4^+^ T cells to produce IL-17 facilitates the process by which this cytokine would be secreted and then bind to its cognate receptor on liver HSCs and liver mDCs, thereby contributing to the pathogenesis of hepatitis B disease. Collectively, the data presented in this study not only provide further insights into the mechanisms underlying HBV pathogenesis, but also suggest potential intervening targets for patient treatment.

## Materials and Methods

### Patients

A total of 166 HBV-infected patients, including 108 patients with CHB and 58 patients with ACLF, were enrolled in this study along with 62 healthy volunteers. CHB and ACLF were diagnosed according to the described criteria [4], [20]. Exclusion criteria were: positive test results for HCV, HDV or human immunodeficiency virus; the presence of any concomitant illness; detection of serological markers of autoimmune disease. All included patients were hospitalized and followed-up in the Department of Infectious Diseases, Southwest Hospital, Third Military Medical University, China from December 2007 to December 2011. The clinical characteristics of these patients and healthy controls are shown in **Table 1**. Written, informed consent was obtained from all subjects prior to participation, and this study was approved by the ethics committee of the Third Military Medical University, Chongqing, China.

**Table 1:** Clinical, viral and immunological criteria for the selection of study subjects.

	CHB	ALCF	Healthy controls
Cases, *n*	108	58	62
Sex, F/M	22/86	9/49	23/39
Age, years	26 (18–46)	32 (20–51)	27 (17–42)
ALT, U/L	126 (53–326)	1829 (360–4526)	24(16–38)
HBsAg, IU/mL	34634.7 (1219.2–138932.7)	38794.2 (1009.2–112093.3)	0
HBeAg-positive, *n*	108	58	0
HBeAb-positive, *n*	0	0	0
HBcAb-positive, *n*	108	58	0
HBV DNA (copies/mL)	331467778 (419622–791520000)	855400 (1028–113090000)	ND
PT, sec	11.19 (9.5–12.6)	31.5 (18.3–42.6)	ND
Recovery, months	>6	>6	NA
Selection criteria	without liver failure	with liver failure	normal

Data values are median (range). PT, prothrombin time; ND, not determined; NA, not applicable.

### Enzyme-linked immunosorbent assay (ELISA)

IL-17, IL-23, IL-8, TNF-α, IL-6 and IL-1β levels were determined by use of the Ready-SET-Go ELISA kit (eBioscience, San Diego, CA, USA), following the manufacturer's instructions.

### Isolation, immunomagnetic cells sorting and culturing of PBMCs and DCs

PBMCs were isolated from 10 mL of heparinized blood by Ficoll-Hypaque density gradient centrifugation. Separation of monocytes and naïve CD4^+^T cells was carried out with immunomagnetic beads (MiltenyiBiotec, Bergisch Gladbach, Germany). DCs were prepared by treating the monocyte population with GM-CSF (500 U/mL) and recombinant human IL-4 for five days, and then treated with HBsAg, mannan, ammonium chloride, chloroquine, Dynasore (Sigma-Aldrich, St. Louis, MO, USA), HBsAg-blocking antibody, or neutralizing anti-MR antibody (clone 19.2, BD). The recombinant HBsAg and HSA were prepared from Chinese Hamster Ovary (*CHO*) cells as previously described [51]. The purity of both HBsAg and HSA reached 99%, as determined by high performance liquid chromatography. HBsAg-blocking antibody (95% purity) was provided by Dr. Qing Mao (Department of Infectious Diseases, Southwestern Hospital, Third Military Medical University, Chongqing, PR China). Co-culture of mDCs and naïve CD4^+^ T cells (1.5∶1) was performed for seven days, and then the expression of IL-17 was detected by FCM or ELISA.

### Flow cytometry assay

The following antibodies used in FCM analysis were from eBioscience: Allophycocyanin-eFluor 780–conjugated anti-CD3; FITC-conjugated anti-CD8; PerCP-conjugated anti-IL-17A; PE-conjugated IL-23R. PBMCs were cultured with 50 ng/mL phorbol 12-myristate 13-acetate (PMA) and 0.5 µg/mL calcium ionophore III (both from Sigma–Aldrich), in the presence of GolgiStop (BD Biosciences Pharmingen, San Jose, CA, USA). Cells were then stained with surface markers, fixed and permeabilized with Cytofix/Cytoperm solution (BD Biosciences Pharmingen), and finally stained with anti-IL-17A. Labeled cells were detected on a FACSAriaI digital cell sorter (BD Biosciences Pharmingen) and data were analyzed by FlowJo v.5.7.2 (TreeStar, Inc., Ashland, OR, USA).

### Total RNA isolation and qPCR

PBMCs and liver tissues were dissolved in TRIzol reagent (Invitrogen, Carlsbad, CA, USA) and total RNA was extracted and subjected to RT-PCR using the PrimeScript RT reagents kit (TaKaRa, Shiga, Japan) according to the manufacturer's directions. qPCR was performed using the SYBR Premix *Ex Taq* polymerase (TaKaRa). Gene-specific primers for human IL-17, IL-23, IL-23R, TNF-α and IL-8 were designed as shown in Table S1. Fluorescence signals were measured after 40 PCR cycles, and all samples were normalized to GAPDH RNA content. All samples were run in duplicate.

### Western blot

Total protein was extracted from liver tissue using T-PER Tissue Protein Extraction Reagent (Pierce, Rockford, IL, USA) and quantified with BCA Protein Assay (Pierce). The proteins were separated by 16% sodium dodecyl sulfate-polyacrylamide gel electrophoresis and transferred onto polyvinylidene fluoride (PVDF) membranes for immunoblotting. The membranes were incubated with goat anti-human IL-17 or IL-17R (R&D Systems, Wiesbaden, Germany), mouse anti-human IL-23p19 (BioLegend, San Diego, CA, USA) or goat anti-human IL-23R (Abcam, San Francisco, CA, USA) for 1 h at room temperature. After incubation with peroxidase-conjugated rabbit anti-goat IgG, or rat anti-mouse IgG for 1 h at room temperature, specific protein bands on the membranes were visualized by the enhanced chemiluminescence method (Amersham, Piscataway, NJ, USA) according to the manufacturer's instructions.

### Immunohistochemistry and immunofluorescence analysis

Liver tissues were embedded in paraffin using the standard protocol. Immunohistochemistry for human IL-17/IL-17R and IL-23/IL-23R was performed as previously described [52] with minor modifications. Briefly, masked antigens were retrieved by microwaving for 20 min in citrate buffer, pH 6.1. After cooling, non-specific binding was blocked by incubation with 5% bovine serum albumin (BSA) in phosphate-buffered saline (PBS) for 30 min. Then, incubation was carried out with the primary antibodies to IL-17 (Santa Cruz Biotechnology, Santa Cruz, CA, USA), IL-23 (BioLegend), IL-23R (Abcam) and IL-17RA (goat anti-human IL-17RA polyclonal IgG; R&D Systems) for 48 h at 4°C in a humidified chamber; after which the slides were incubated with the secondary, biotin-conjugated antibody, followed by sequential incubation with horseradish peroxidase-streptavidin and the peroxidase substrate 3′-diaminobenzidine (DAB). Slides were then counterstained with hematoxylin.

For the double-staining of anti-IL-17 plus anti-CD4, anti-γδTCR, anti-MPO, or anti-IL-23R, anti-IL-23p19 plus anti-CD11c, anti-CD303, CD68, or anti-hepatocyte and anti-IL-17RA plus anti-α-SMA, anti-IL-17, anti-IL-23p19 antibody, the first antibody (1∶100) was applied and slides incubated for 48 h at 4°C in a humidified chamber. The second antibody anti-human CD4, δTCR, MPO, IL-23R, CD11c, CD303, CD68, Hepatocyte or α-SMA (1∶100 in PBS containing 5% BSA) was then applied for overnight at 4°C. TRITC-, FITC- or CY5-labeled anti-goat IgG (1∶500 in PBS) and Dylight488-labeled anti-mouse or anti-rabbit IgG (diluted 1∶200; Zhongshan Goldenbridge Biotechnology, Beijing, China) were applied for 60 min at 37°C. Nuclear counterstaining was performed by incubating slides for 5 min with diaminidophenylindol (DAPI, 1∶100 in PBS; Invitrogen). Normal mouse, goat or rabbit IgG was used as a negative control. Images were obtained by the digital confocal laser scanning system MRC-600 (Bio-Rad, Hercules, CA, USA).

### HSC manipulations

The commercial-source human primary HSCs isolated from healthy human liver (ScienCell, San Diego, CA, USA) or the freshly prepared primary HSCs from liver tissue of ACLF patients who received surgical liver transplantation [53] were resuspended in HSC medium (DMEM+10% fetal bovine serum (FBS; Invitrogen)+1% penicillin/streptomycin) and plated on a 24 well non-tissue culture-treated plate. After overnight incubation, fresh culture solution with 5 µg/mL of IL-17R-blocking antibody or 10 ng/mL of rhIL-17 was added and the HSCs were cultured for an additional 48 h. The supernatants were subjected to ELISA to measure the levels of secreted cytokines IL-6, IL-8, TNF-α, and IL-1β.

### Virological assessment

The virological assay was performed as previously described [54].

### Statistical analysis

Comparisons between various groups were performed using the Mann-Whitney *U t*est. Results are expressed as the mean ± standard error of the mean (SEM), unless noted otherwise. Correlations between variables were evaluated using the Spearman's rank correlation test. For all tests, two-sided *P*<0.05 was considered significant.

## Supporting Information

Figure S1 **Elevated IL-17 expression in HBV-infected liver tissue.** Relative mRNA and protein expressions of IL-17 in liver tissues of hepatitis B patients and healthy controls were determined by (**A**) qPCR and (**B**) Western blot assays, respectively. Error bars indicate SD. *P<0.05; **P<0.01. (**C**) Expression of IL-17 in liver tissue was detected by immunohistochemical staining (magnification 100×). (**D**) Expression of IL-17 mRNA in PBMC or liver biopsy tissue from the identical hepatitis B patients.
(TIF)

Figure S2 **IL-17 is derived from CD4^+^T cells but not γδT cells or neutrophils in liver tissues of hepatitis B patients.** Frozen liver biopsy sections from CHB patients were stained with fluorescent-labeled antibodies. Co-localization of IL-17 (red) with (**A**) CD4 (green, CD4^+^ T cells), (**B**) γδTCR (green, a unique marker of the γδT subset) or (**C**) MPO (green, a marker of neutrophils) is shown. The right panels are enlarged images of the area in the left panel that is demarcated by a white dashed-line. MPO, myeloperoxidase. (magnification 100×).
(TIF)

Figure S3 **HBsAg in the serum of hepatitis B patients induced IL-23 production in macrophages.** Monocytes were isolated from PBMCs of healthy blood donors and macrophages were induced by culturing in the presence of GM-CSF for five days. Then, the macrophages were stimulated by exposure to 50 µL HC serum or 50 µL ACLF serum (HBsAg concentration: 79876.4 IU/mL, equivalent to 8 µg/mL) in a 200 µL culture system. For the blocking group, the ACLF serum had been pretreated with 50 µg/mL of HBsAg antibody for 30 min. ELISA was used to detect the concentration of IL-23 in the supernatants. The data represent one of three independent experiments with similar results. Error bars indicate SD. **P<0.01 vs. the HC serum group.
(TIF)

Figure S4 **Endocytosis-independent IL-23 production from DCs stimulated by HBcAg.** (**A**) Monocytes were isolated from PBMCs of healthy blood donors and mDCs were induced by five days of culturing in the presence of GM-CSF (50 ng/mL) and recombinant human IL-4 (5 ng/mL). mDCs were then stimulated by HBcAg alone or in the presence of ammonium chloride (10 µM), chloroquine (10 mM), Dynasore (80 µM), or HBcAg-blocking antibody (10 µg/mL) for 40 hours. FCM was used to detect the production of IL-23 in the mDCs (CD11c positive). The data represent one of three independent experiments with similar results. (**B**) Pooled data indicate the percentages of IL-23^+^ cells within the CD11c^+^ cell population. Error bars indicate SD.
(TIF)

Figure S5 **Effects of chloroquine on the function of mDCs.** (**A**) Monocytes were isolated from PBMCs of healthy blood donors and mDCs were induced by culturing for five days in the presence of GM-CSF (50 ng/mL) and recombinant human IL-4 (5 ng/mL). Then, mDCs were stimulated by 24 hours of exposure to LPS alone or together with chloroquine (10 mM). FCM was used to detect the co-stimulatory molecules on mDCs. The data represent one of three independent experiments with similar results. (**B**) Pooled data indicate the ratio of mean fluorescence intensity (MFI) of co-stimulatory molecules on mDCs stained by specific antibody to MFI of co-stimulatory molecules on mDCs stained by an isotype of the specific antibody. Error bars indicate SD.
(TIF)

Figure S6 **IL-23 is indispensable for HBV antigen-stimulated IL-17 production and Th17 differentiation.** The mDCs and naïve CD4^+^ T cells from PBMCs of healthy blood donors (1.5∶1) were cultured for seven days in the presence of HBsAg (2 µg/mL), IL-23-blocking antibody (500 µg/mL) or rhIL-23 (20 ng/mL). The concentration of secreted IL-17 in the supernatant was detected by ELISA (**A**), and IL-17^+^CD4^+^ T cells were analyzed by FCM assay (**B**). The data represent one of three independent experiments with similar results. Error bars indicate SD. **P<0.01 *vs.* the HBsAg group.
(TIF)

Figure S7 **Expression of IL-17 receptor in liver tissue.** (**A**) Protein expression of IL-17R detected by Western blotting of liver tissues from healthy controls and patients with hepatitis B. Error bars indicate SD. *P<0.05; **P<0.01. (**B**) The expression of IL-17R in liver tissues from CHB patients detected by immunohistochemical staining (magnification 100×). (**C**) and (**D**) Co-localization of IL-17R (red;), α-SMA (green, an HSC unique marker) or CD11c (green) in liver tissue from CHB patients detected by confocal fluorescence microscopy by using anti-human IL-17 RA polyclonal antibody, anti-α-SMA mAb or anti-CD11c mAb as primary antibody, respectively.
(TIF)

Table S1 **Primers designed for detection of target genes.**
(DOC)
