# Supplementary figures and images for: Hepatitis B Virus Induces IL-23 Production in Antigen Presenting Cells and Causes Liver Damage via the IL-23/IL-17 Axis
Source: PLoS Pathog. 2013 Jun 27;9(6):e1003410. doi: 10.1371/journal.ppat.1003410 (PMC3694858; doi:10.1371/journal.ppat.1003410)

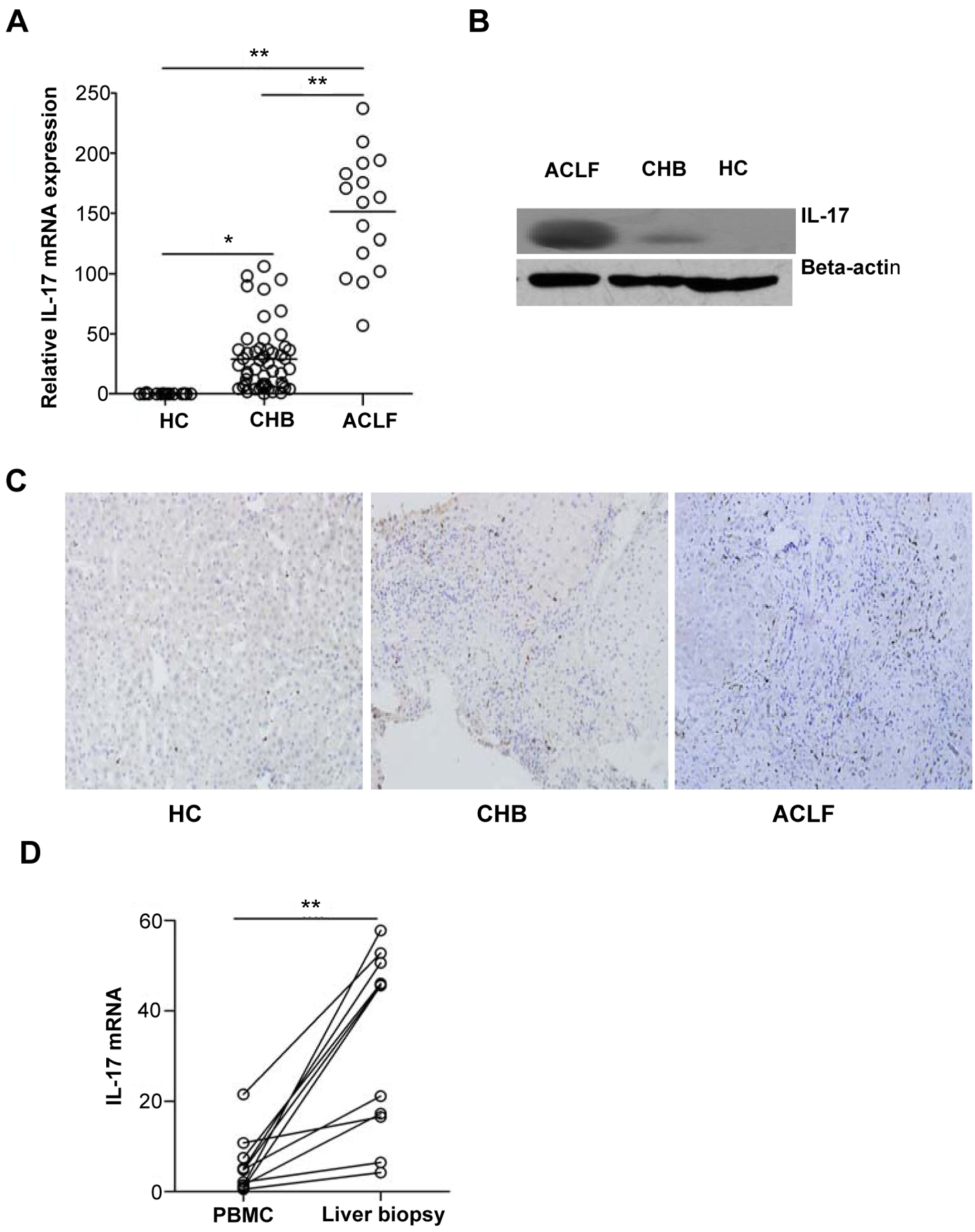

Supplement: Figure S1 — Elevated IL-17 expression in HBV-infected liver tissue. Relative mRNA and protein expressions of IL-17 in liver tissues of hepatitis B patients and healthy controls were determined by (A) qPCR and (B) Western blot assays, respectively. Error bars indicate SD. *P<0.05; **P<0.01. (C) Expression of IL-17 in liver tissue was detected by immunohistochemical staining (magnification 100×). (D) Expression of IL-17 mRNA in PBMC or liver biopsy tissue from the identical hepatitis B patients. (TIF) [file ppat.1003410.s001.tif]

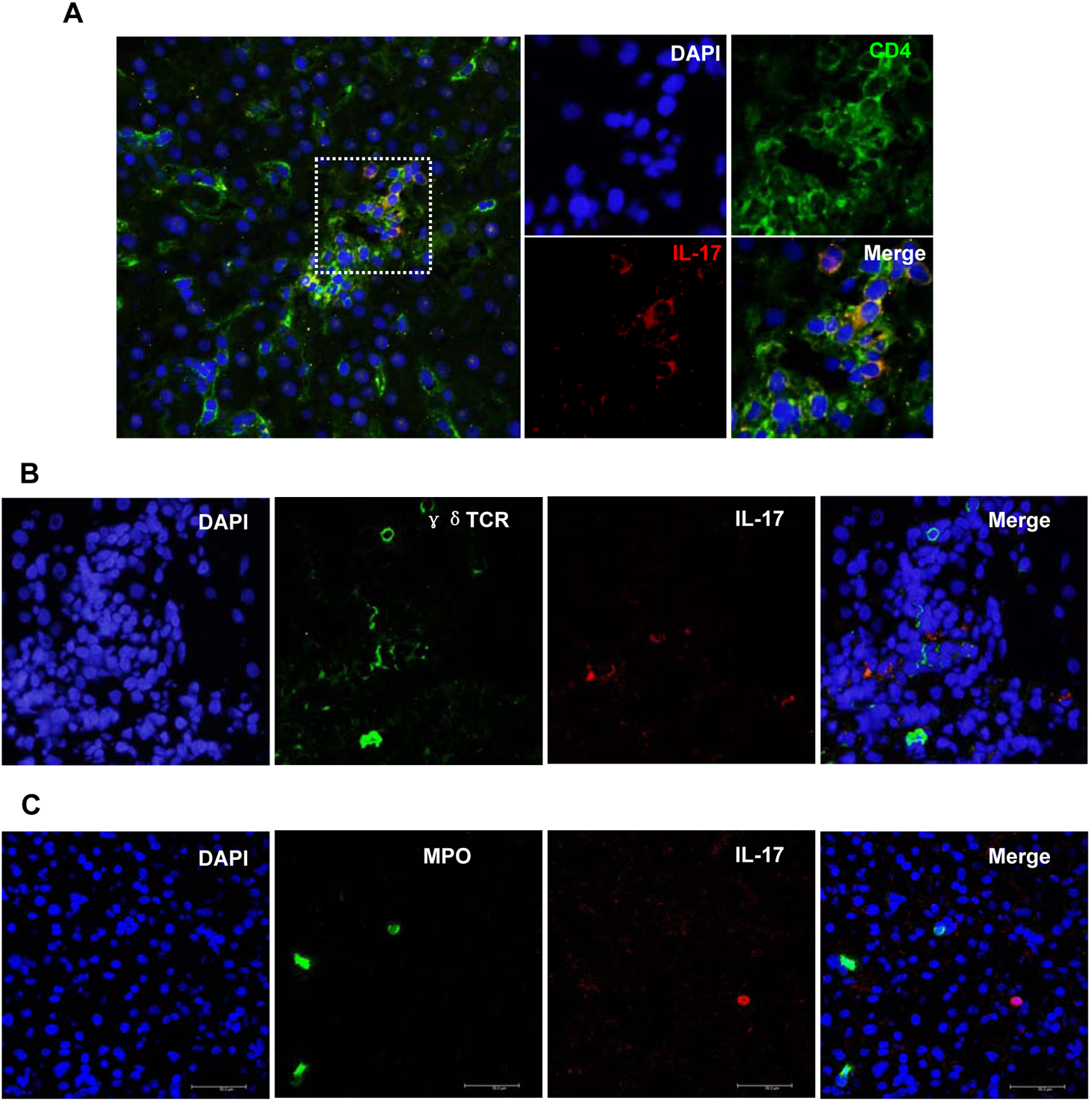

Supplement: Figure S2 — IL-17 is derived from CD4+T cells but not γδT cells or neutrophils in liver tissues of hepatitis B patients. Frozen liver biopsy sections from CHB patients were stained with fluorescent-labeled antibodies. Co-localization of IL-17 (red) with (A) CD4 (green, CD4+ T cells), (B) γδTCR (green, a unique marker of the γδT subset) or (C) MPO (green, a marker of neutrophils) is shown. The right panels are enlarged images of the area in the left panel that is demarcated by a white dashed-line. MPO, myeloperoxidase. (magnification 100×). (TIF) [file ppat.1003410.s002.tif]

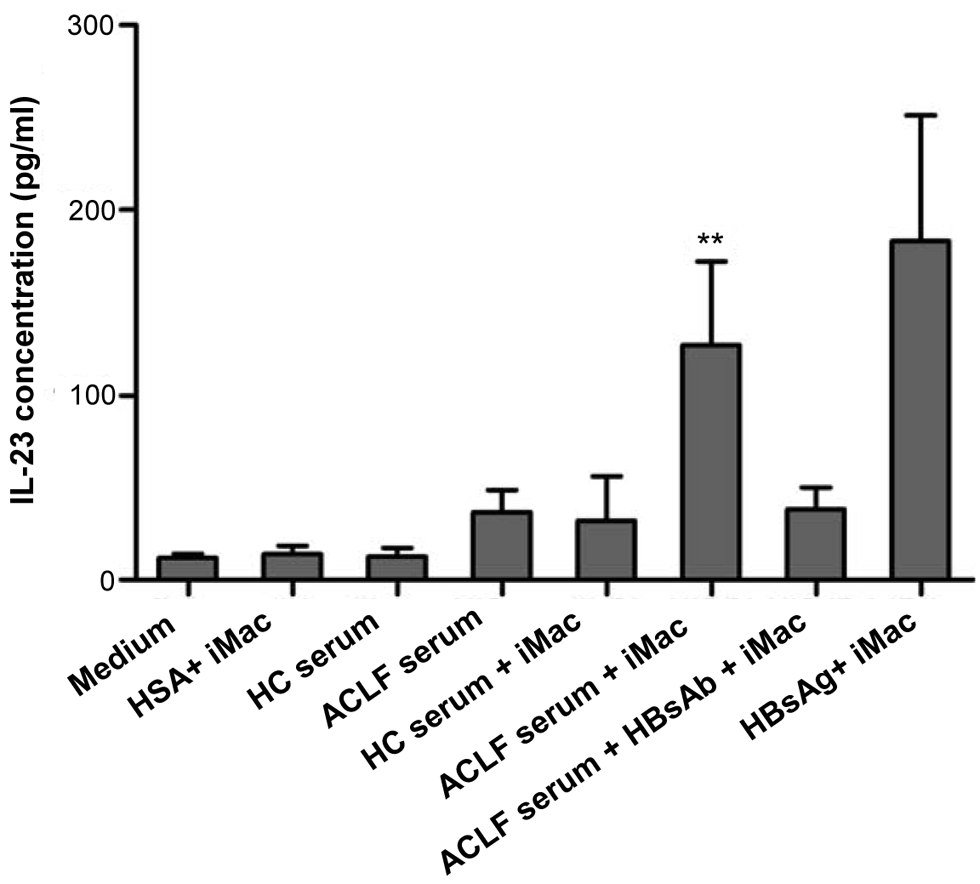

Supplement: Figure S3 — HBsAg in the serum of hepatitis B patients induced IL-23 production in macrophages. Monocytes were isolated from PBMCs of healthy blood donors and macrophages were induced by culturing in the presence of GM-CSF for five days. Then, the macrophages were stimulated by exposure to 50 µL HC serum or 50 µL ACLF serum (HBsAg concentration: 79876.4 IU/mL, equivalent to 8 µg/mL) in a 200 µL culture system. For the blocking group, the ACLF serum had been pretreated with 50 µg/mL of HBsAg antibody for 30 min. ELISA was used to detect the concentration of IL-23 in the supernatants. The data represent one of three independent experiments with similar results. Error bars indicate SD. **P<0.01 vs. the HC serum group. (TIF) [file ppat.1003410.s003.tif]

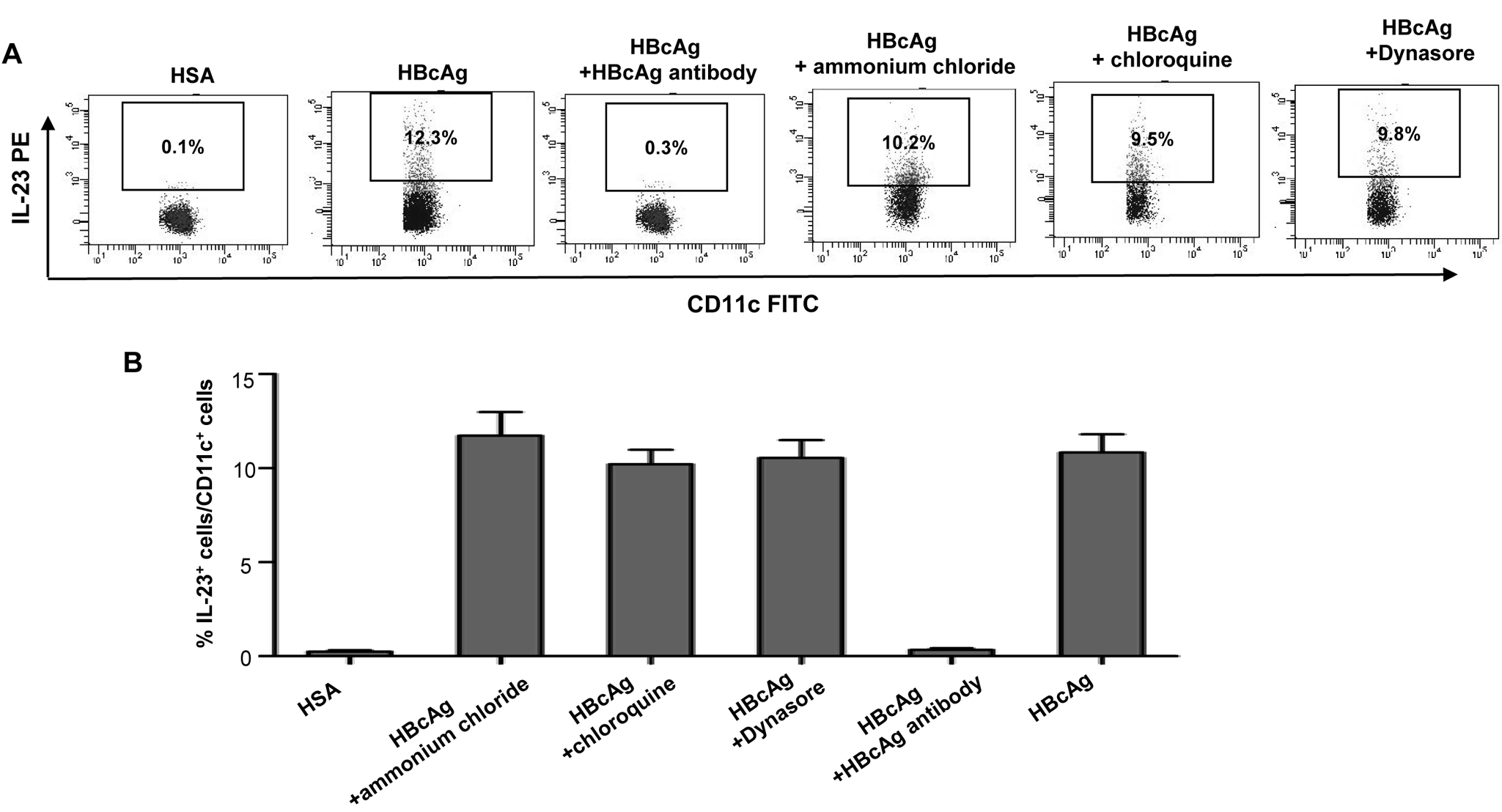

Supplement: Figure S4 — Endocytosis-independent IL-23 production from DCs stimulated by HBcAg. (A) Monocytes were isolated from PBMCs of healthy blood donors and mDCs were induced by five days of culturing in the presence of GM-CSF (50 ng/mL) and recombinant human IL-4 (5 ng/mL). mDCs were then stimulated by HBcAg alone or in the presence of ammonium chloride (10 µM), chloroquine (10 mM), Dynasore (80 µM), or HBcAg-blocking antibody (10 µg/mL) for 40 hours. FCM was used to detect the production of IL-23 in the mDCs (CD11c positive). The data represent one of three independent experiments with similar results. (B) Pooled data indicate the percentages of IL-23+ cells within the CD11c+ cell population. Error bars indicate SD. (TIF) [file ppat.1003410.s004.tif]

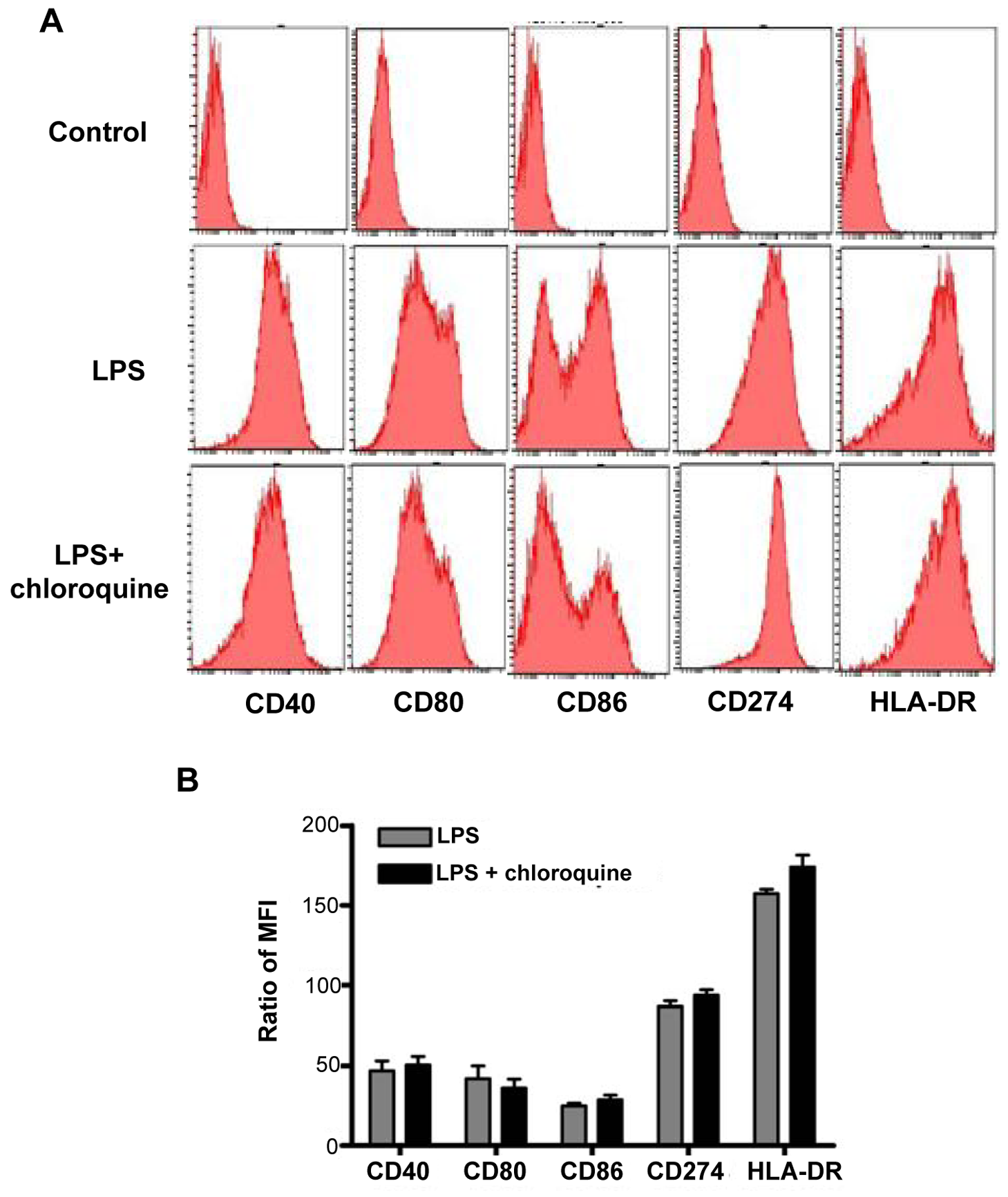

Supplement: Figure S5 — Effects of chloroquine on the function of mDCs. (A) Monocytes were isolated from PBMCs of healthy blood donors and mDCs were induced by culturing for five days in the presence of GM-CSF (50 ng/mL) and recombinant human IL-4 (5 ng/mL). Then, mDCs were stimulated by 24 hours of exposure to LPS alone or together with chloroquine (10 mM). FCM was used to detect the co-stimulatory molecules on mDCs. The data represent one of three independent experiments with similar results. (B) Pooled data indicate the ratio of mean fluorescence intensity (MFI) of co-stimulatory molecules on mDCs stained by specific antibody to MFI of co-stimulatory molecules on mDCs stained by an isotype of the specific antibody. Error bars indicate SD. (TIF) [file ppat.1003410.s005.tif]

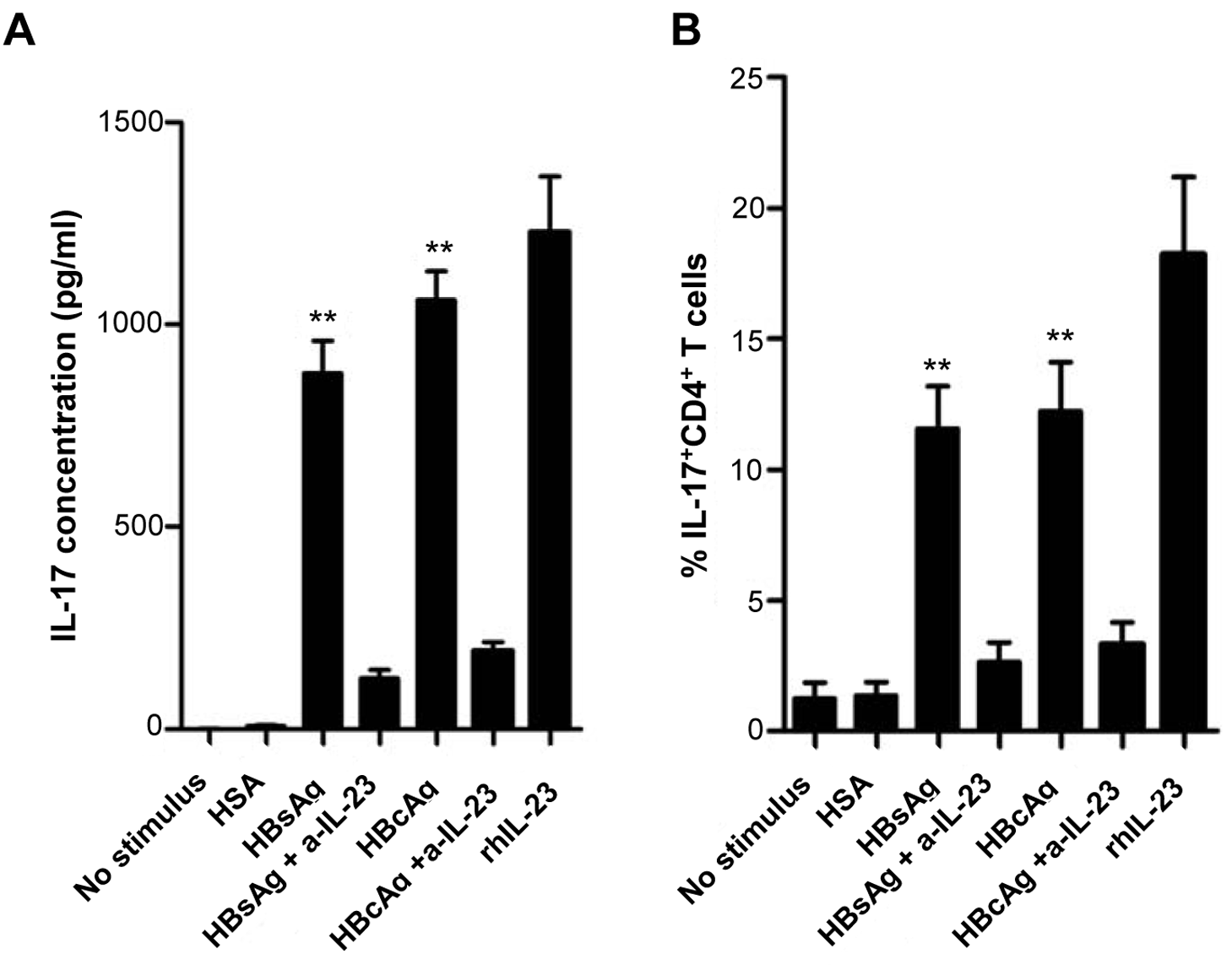

Supplement: Figure S6 — IL-23 is indispensable for HBV antigen-stimulated IL-17 production and Th17 differentiation. The mDCs and naïve CD4+ T cells from PBMCs of healthy blood donors (1.5∶1) were cultured for seven days in the presence of HBsAg (2 µg/mL), IL-23-blocking antibody (500 µg/mL) or rhIL-23 (20 ng/mL). The concentration of secreted IL-17 in the supernatant was detected by ELISA (A), and IL-17+CD4+ T cells were analyzed by FCM assay (B). The data represent one of three independent experiments with similar results. Error bars indicate SD. **P<0.01 vs. the HBsAg group. (TIF) [file ppat.1003410.s006.tif]

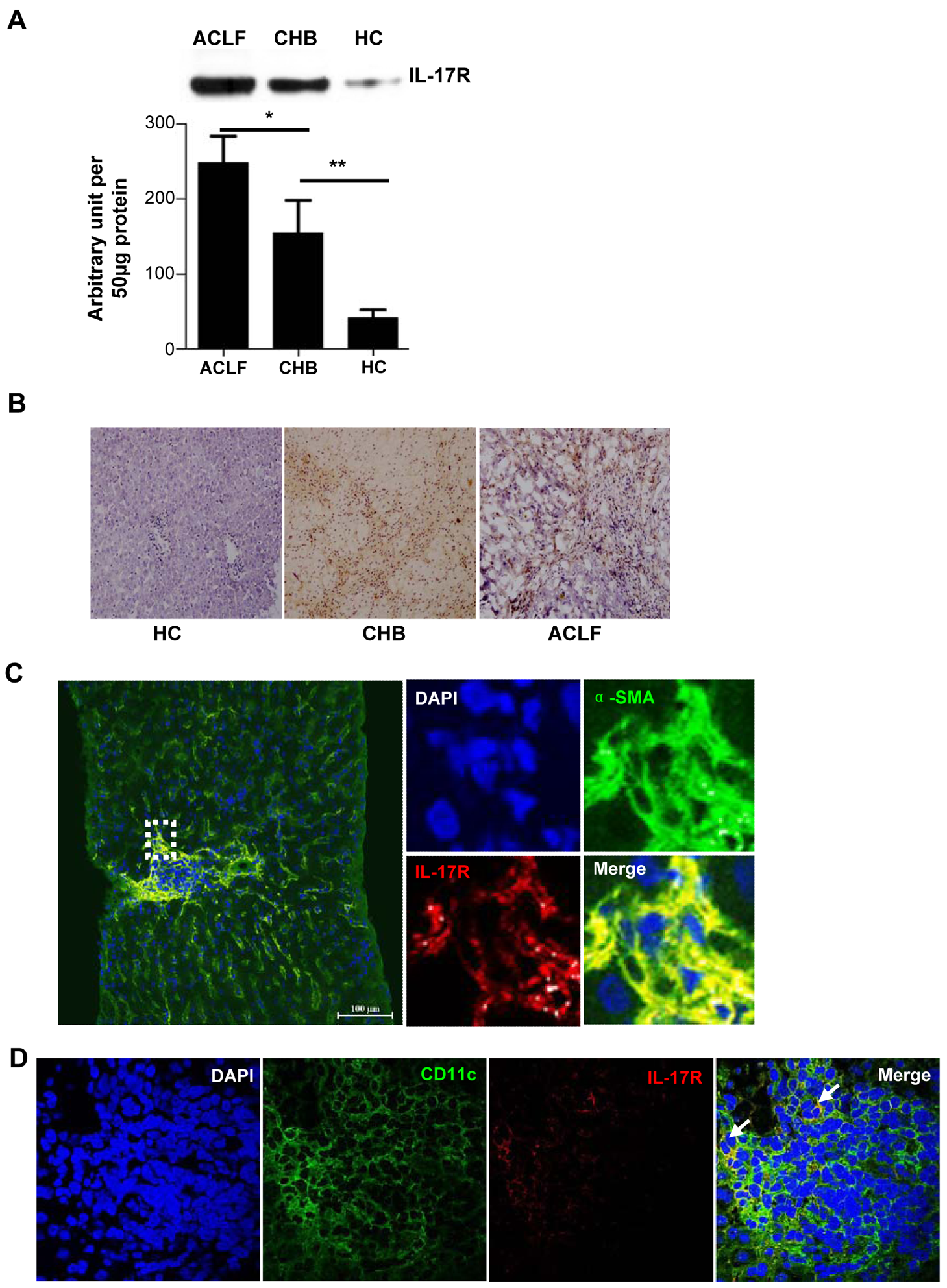

Supplement: Figure S7 — Expression of IL-17 receptor in liver tissue. (A) Protein expression of IL-17R detected by Western blotting of liver tissues from healthy controls and patients with hepatitis B. Error bars indicate SD. *P<0.05; **P<0.01. (B) The expression of IL-17R in liver tissues from CHB patients detected by immunohistochemical staining (magnification 100×). (C) and (D) Co-localization of IL-17R (red;), α-SMA (green, an HSC unique marker) or CD11c (green) in liver tissue from CHB patients detected by confocal fluorescence microscopy by using anti-human IL-17 RA polyclonal antibody, anti-α-SMA mAb or anti-CD11c mAb as primary antibody, respectively. (TIF) [file ppat.1003410.s007.tif]
