# Supplementary material for: Hepatitis B Virus Induces IL-23 Production in Antigen Presenting Cells and Causes Liver Damage via the IL-23/IL-17 Axis
Source: PLoS Pathog. 2013 Jun 27;9(6):e1003410. doi: 10.1371/journal.ppat.1003410 (PMC3694858; doi:10.1371/journal.ppat.1003410)
Supplement: Table S1 — Primers designed for detection of target genes. (DOC) [file ppat.1003410.s008.doc]

**Table S**1. Primers designed for detection of target genes

| **Gene** | **Forward primer, 5′-3′** | **Reverse primer, 5′-3′** |
| --- | --- | --- |
| IL-17 | TCAACCCGATTGTCCACCAT | GAGTTTAGTCCGAAATGAGGCTG |
| IL-23 | GCTTCAAAATCCTTCGCAG | TATCTGAGTGCCATCCTTGAG |
| IL-23R | ATAATTCCAGTGAGCAGGTCC | TTCTTGTAGTCTGTAGGCTTGTG |
| IL-8 | TTCTAGGACAAGAGCCAGGAAG | GGGTGGAAAGGTTTGGAGTATG |
| TNF-α | ATGAGCACTGAAAGCATGATCC | GAGGGCTGATTAGAGAGAGGTC |
